# Supplementary figures and images for: Gene regulatory network structure informs the distribution of perturbation effects
Source: PLoS Comput Biol. 2025 Sep 2;21(9):e1013387. doi: 10.1371/journal.pcbi.1013387 (PMC12419648; doi:10.1371/journal.pcbi.1013387)

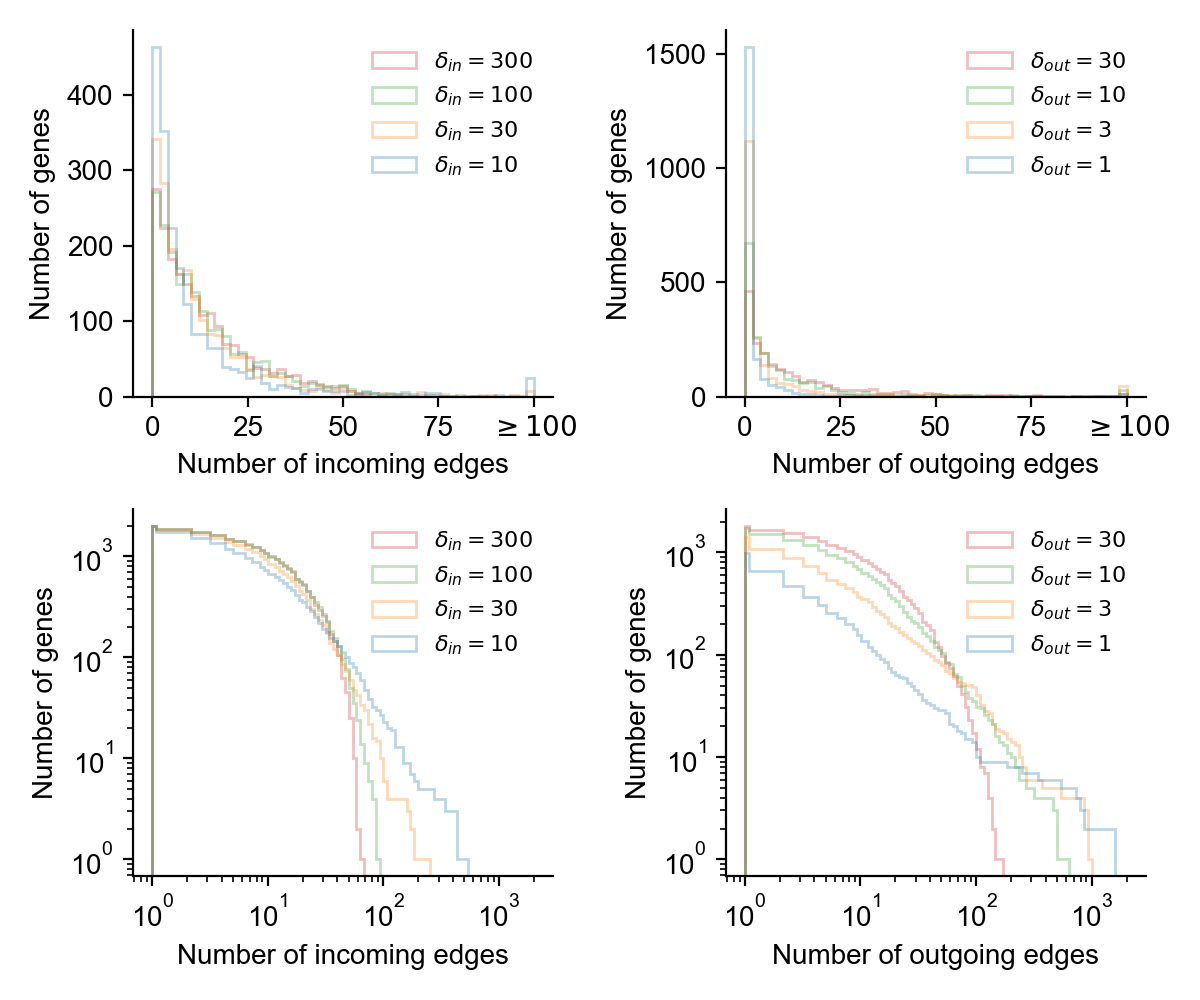

Supplement: S1 Fig — Example in- and out-degree distribution for four of the 1,920 GRNs simulated for the study. The networks have n=2,000 genes, r = 16, k = 1, w = 1, and δin and δout either equal to 10 or as specified by the subpanel legend. The left columns show the relationship between δin and the distribution of incoming edges per gene, and the right columns show the relationship between δout and the distribution of outgoing edges per gene. The top and bottom rows display different views of the same data; the x-axis values in the top row are soft clipped at 100, and the x- and y-axes in the bottom row are log-scaled. (TIFF) [file pcbi.1013387.s001.tiff]

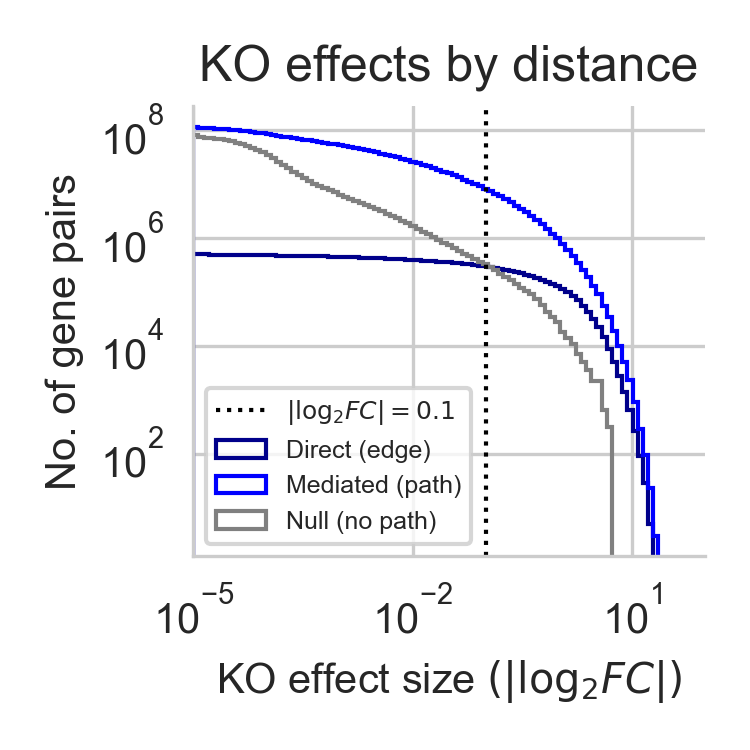

Supplement: S2 Fig — Same as Fig 3D, but with distances binned by whether pairs of genes are connected by an edge (distance 1, a “direct effect”), any path (distance greater than 1, a “mediated effect”), or no path at all (“null”). Note also that the y-axis is the count of gene pairs with a perturbation effect of at least the magnitude given on the x-axis—that is, the distribution shown is a non-normalized inverse CDF. Gene pairs are pooled from the 50 example GRNs in Fig 3. (TIFF) [file pcbi.1013387.s002.tiff]

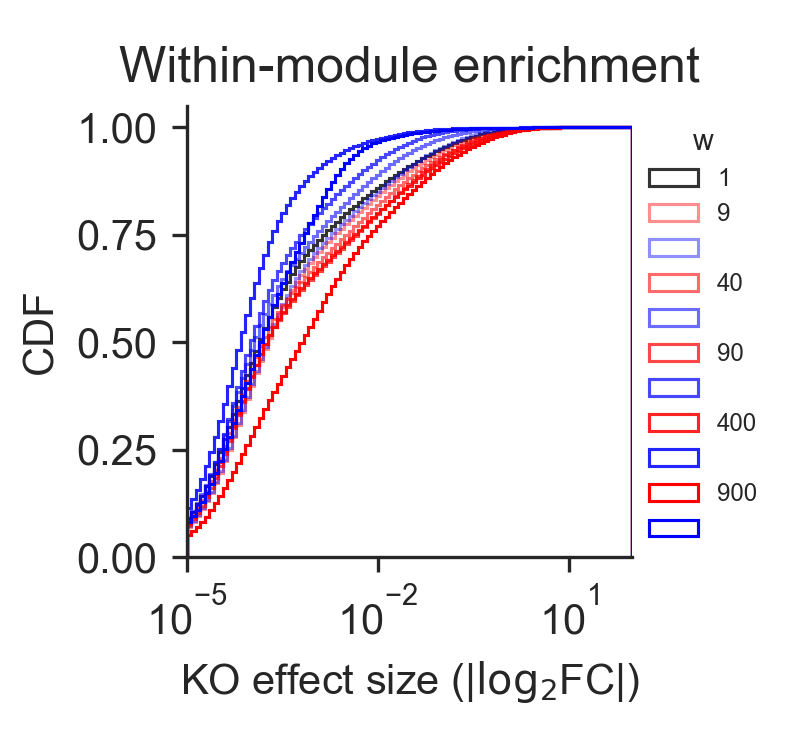

Supplement: S3 Fig — Same as Fig 3E, with within-module perturbation effects in red and between-module perturbation effects in blue. Here, networks are chosen so as to highlight the effect of the modularity term w. Each pair of blue and red tracelines is distribution of the within- (red) or across-module perturbation effects a single GRN. The generating parameters for these GRNs vary w (see legend) but hold other parameters constant, as follow: p = 1/4, k = 50, δin=10, δout=10. (TIFF) [file pcbi.1013387.s003.tiff]

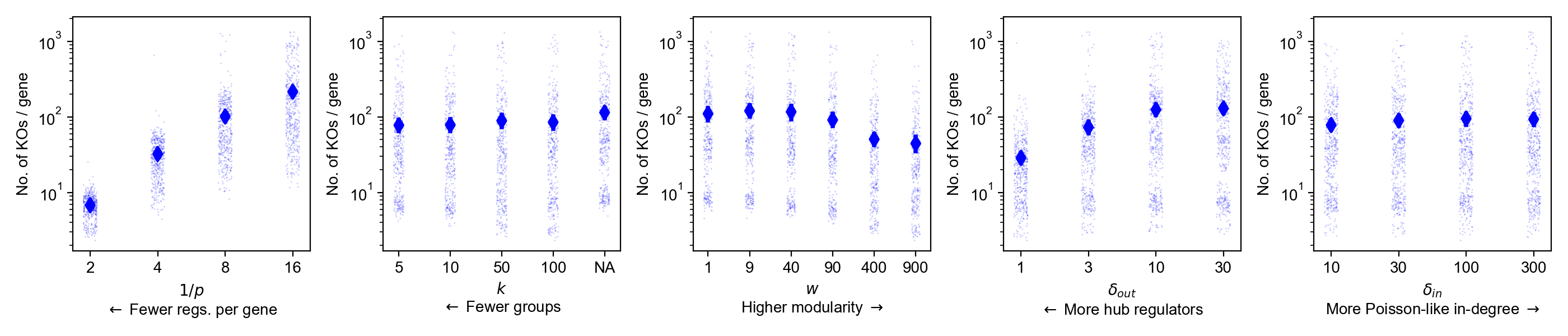

Supplement: S4 Fig — Counts of the number of perturbation effects per gene in the GRN with |log2FC |≥0.1 in each synthetic GRN, as a function of network generating parameters. Each panel shows all 1,920 GRNs as individual points, stratified by parameter values. Each distribution is annotated with its mean over GRNs (diamond points). We observe a similar direction of effect for each parameter as with the statistics presented in Fig 4. (TIFF) [file pcbi.1013387.s004.tiff]

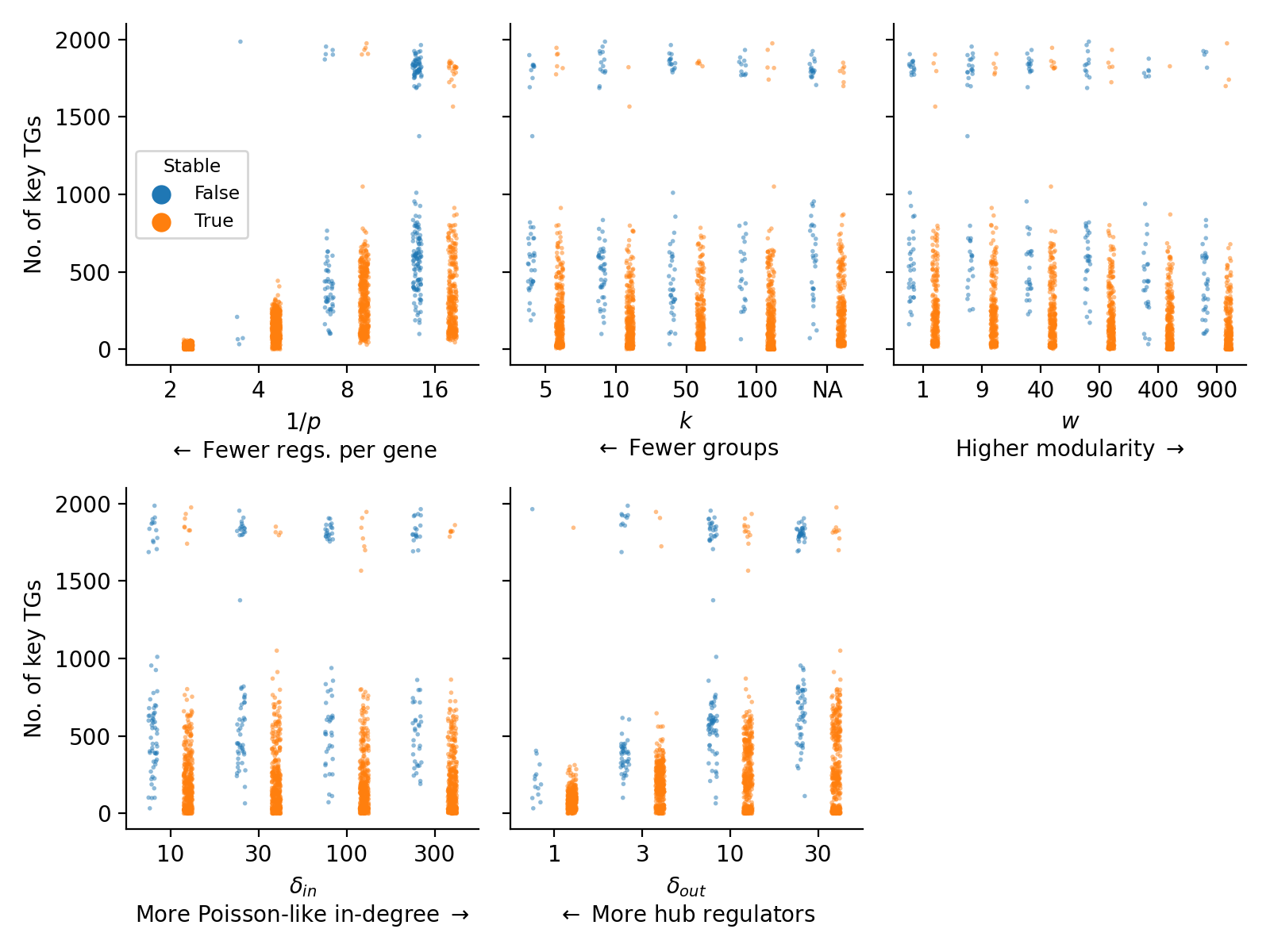

Supplement: S5 Fig — Counts of the number of genes which are hub targets in each synthetic GRN, as a function of network generating parameters, and stratifying by whether the expression equilibrium point of the synthetic GRN is stable (Methods). In all, 1,693 of the 1,920 GRNs (88.2%) reach an expression equilibrium through forward simulation of the SDE which is a stable fixed point of the corresponding ODE. These GRNs tend to be sparse (lower 1/p), modular (higher w), and have more hub regulators (lower δout), consistent with the direction of effect on the number of strong KOs and key target genes (as in Fig 4). (TIFF) [file pcbi.1013387.s005.tiff]

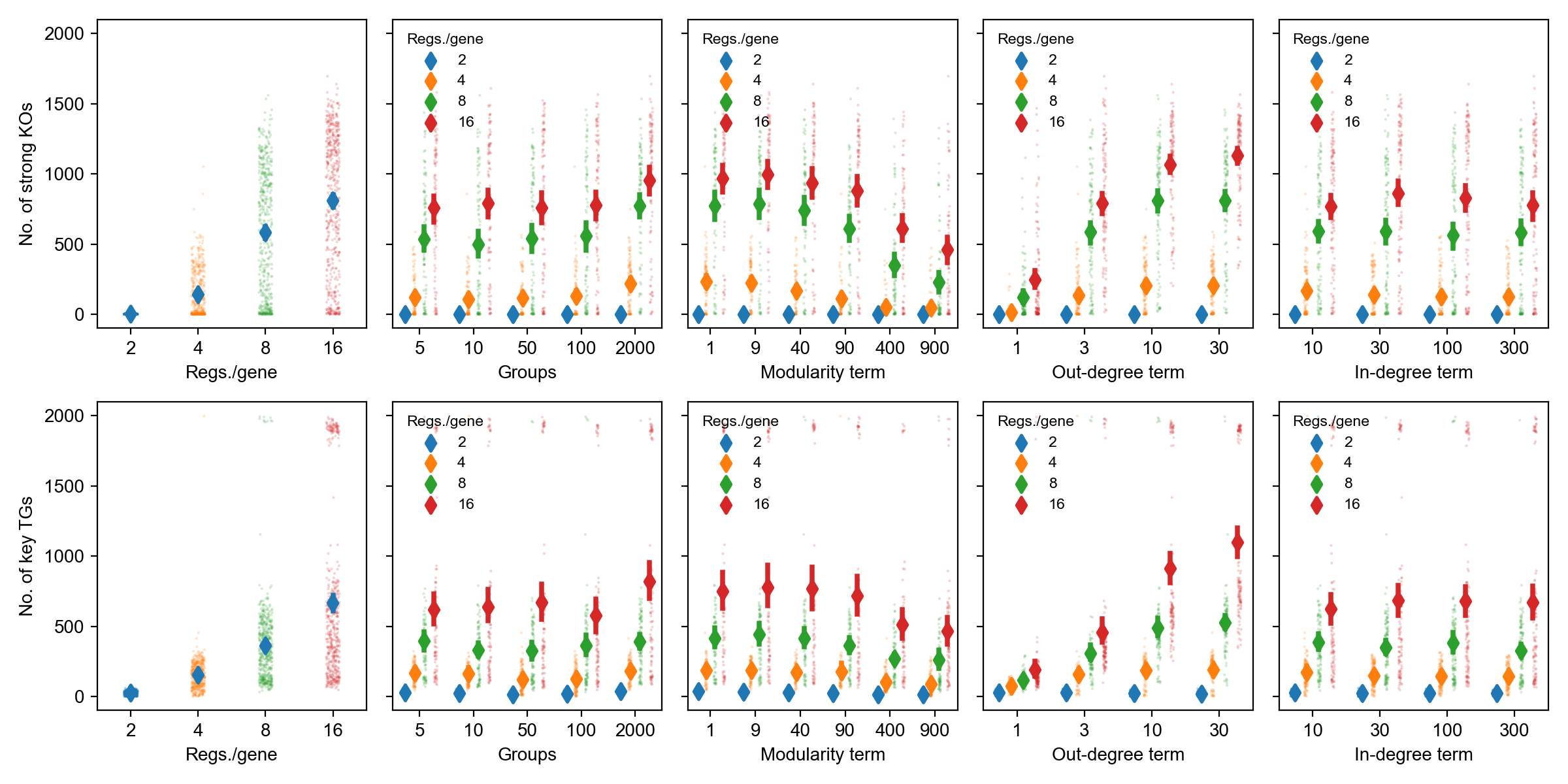

Supplement: S6 Fig — Counts of the number of genes which are strong knockouts (KOs, top row) and key target genes (bottom row) in each synthetic GRN. Each panel shows all 1,920 GRNs as individual points, across values of network generating parameters (x-axes), with additional stratification by the sparsity term 1/p. Each distribution is annotated with its mean over GRNs in each bin (diamond points). There is no obvious visual evidence for interactions between the parameters. (TIFF) [file pcbi.1013387.s006.tiff]

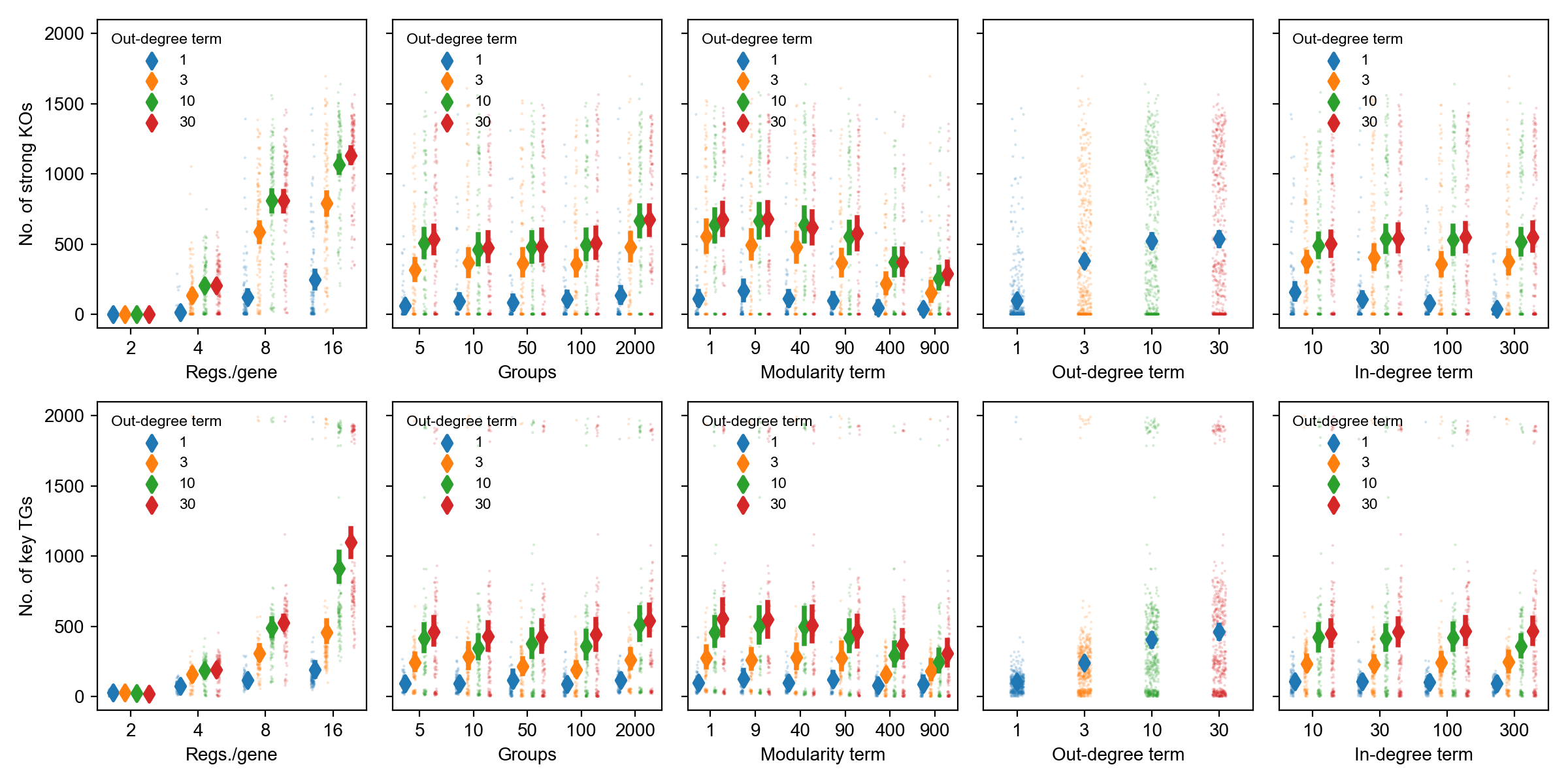

Supplement: S7 Fig — Counts of the number of genes which are strong knockouts (KOs, top row) and key target genes (bottom row) in each synthetic GRN. Each panel shows all 1,920 GRNs as individual points, across values of network generating parameters (x-axes), with additional stratification by the out-degree term δout. Each distribution is annotated with its mean over GRNs in each bin (diamond points). There is no obvious visual evidence for interactions between the parameters. (TIFF) [file pcbi.1013387.s008.tiff]

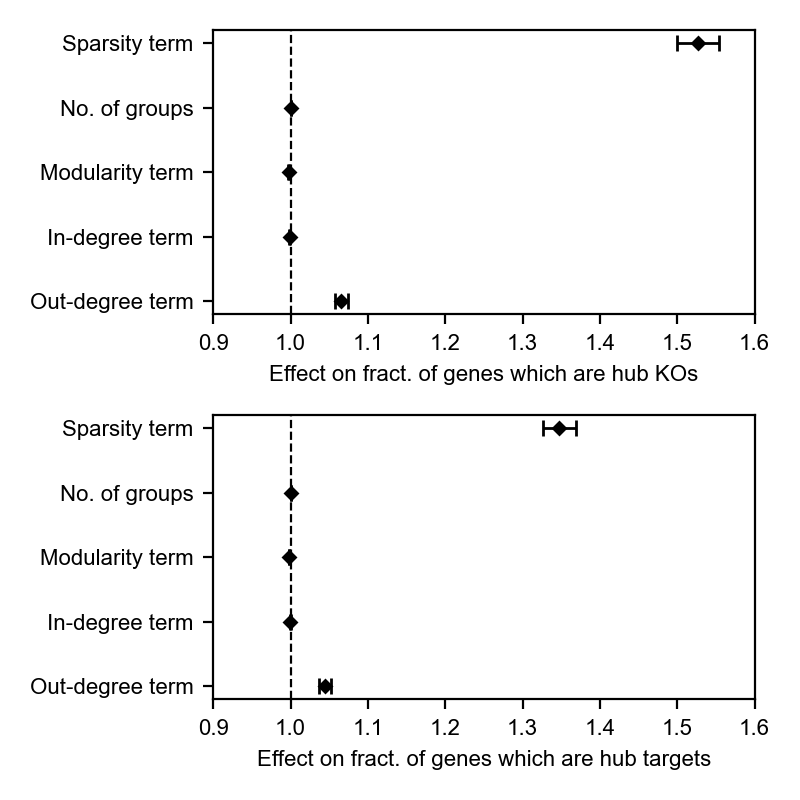

Supplement: S8 Fig — Coefficients from regressing the logit-transformed fraction of genes which are hub knockouts (top) or target genes (bottom) on network generating parameters. Error bars denote 95% confidence intervals for the regression coefficients. Model summaries can be found in S1 Table (hub knockouts) and in S2 Table (target genes). (TIFF) [file pcbi.1013387.s009.tiff]

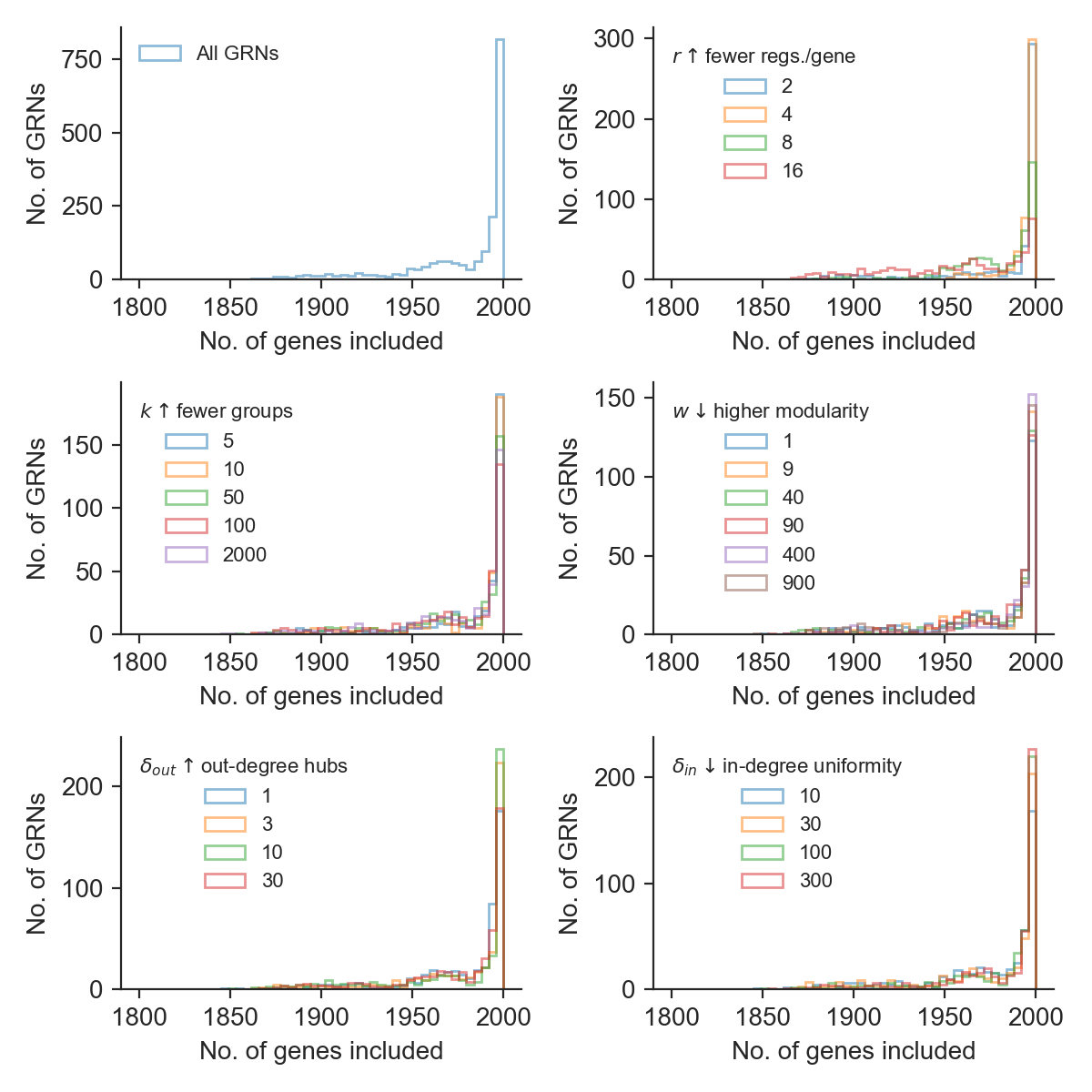

Supplement: S9 Fig — Distribution of the number of non-lowly expressed genes included in the matching analysis in Fig 5 (see Methods). The top left panel shows the distribution over all 1,920 GRNs; each other subpanel shows the conditional distributions for each generating parameter. We note an interaction between the number of regulators per gene and the number of genes included in downstream analysis. (TIFF) [file pcbi.1013387.s012.tiff]

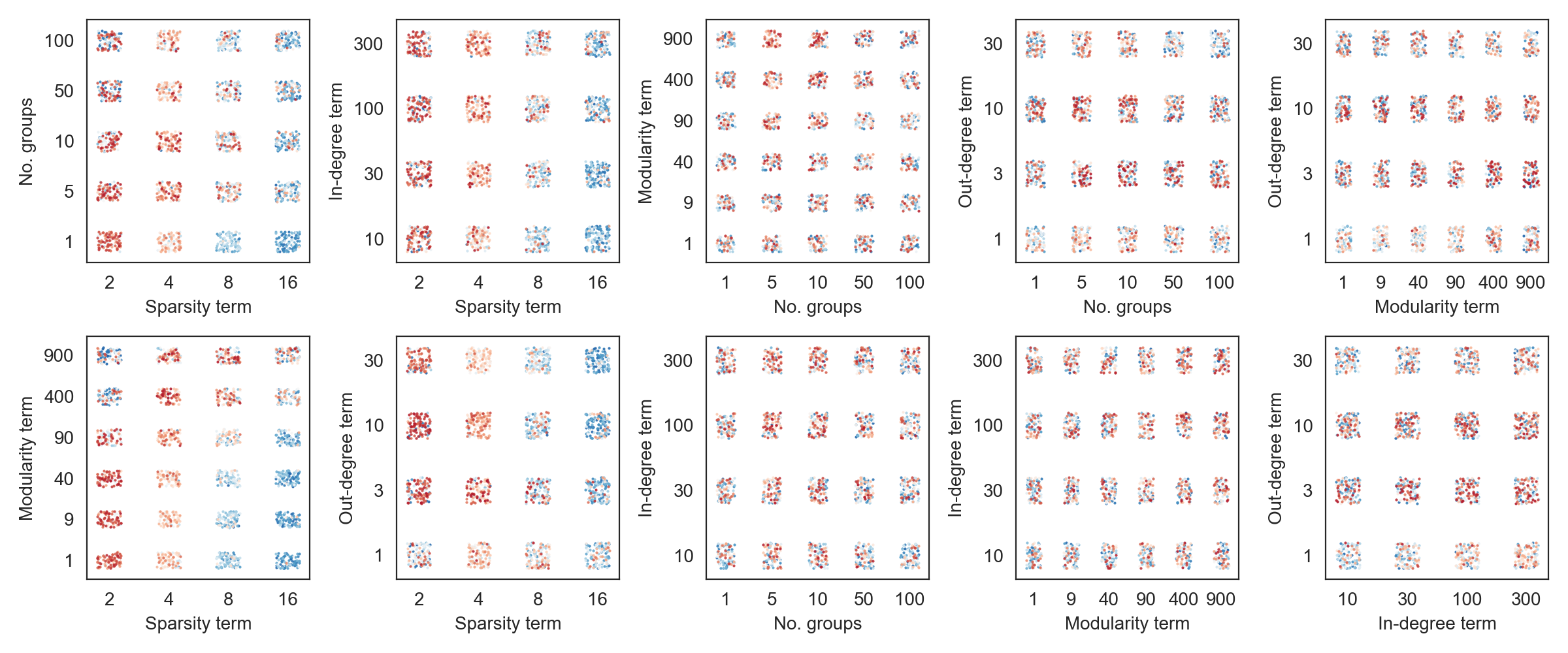

Supplement: S10 Fig — As in Fig 5C–E, we show the relationship between pairs of network generating parameters and goodness of fit to the cumulative distribution of perturbation effects from experimental Perturb-seq data. Each GRN (one point in every subpanel) is colored by its ranked fit to data: the synthetic GRNs are ranked separately by Kolmogorov-Smirnov p-value for incoming and outgoing perturbation effects, then the sum of these two ranks is used to produce an overall ranking. Intense red color indicates better ranked fit to data, and intense blue color indicates a worse ranking. (TIFF) [file pcbi.1013387.s013.tiff]

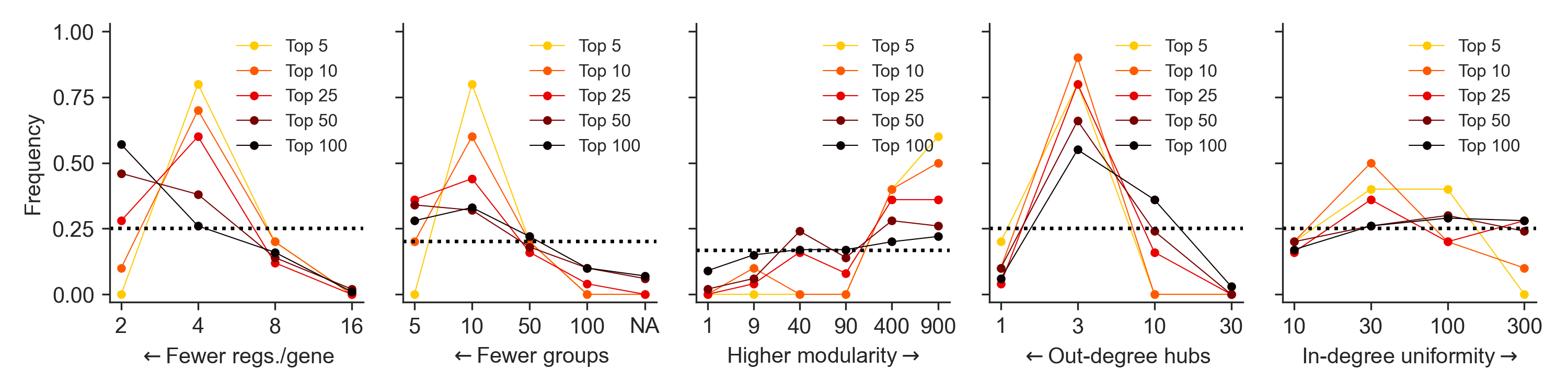

Supplement: S11 Fig — Replication of Fig 5C–G, changing the number of plotted GRNs. Distribution of network generating parameters for the k GRNs that are best matched to Perturb-seq data (by K-S p-value rank for the number of incoming and outgoing effects; see Methods). (TIFF) [file pcbi.1013387.s014.tiff]

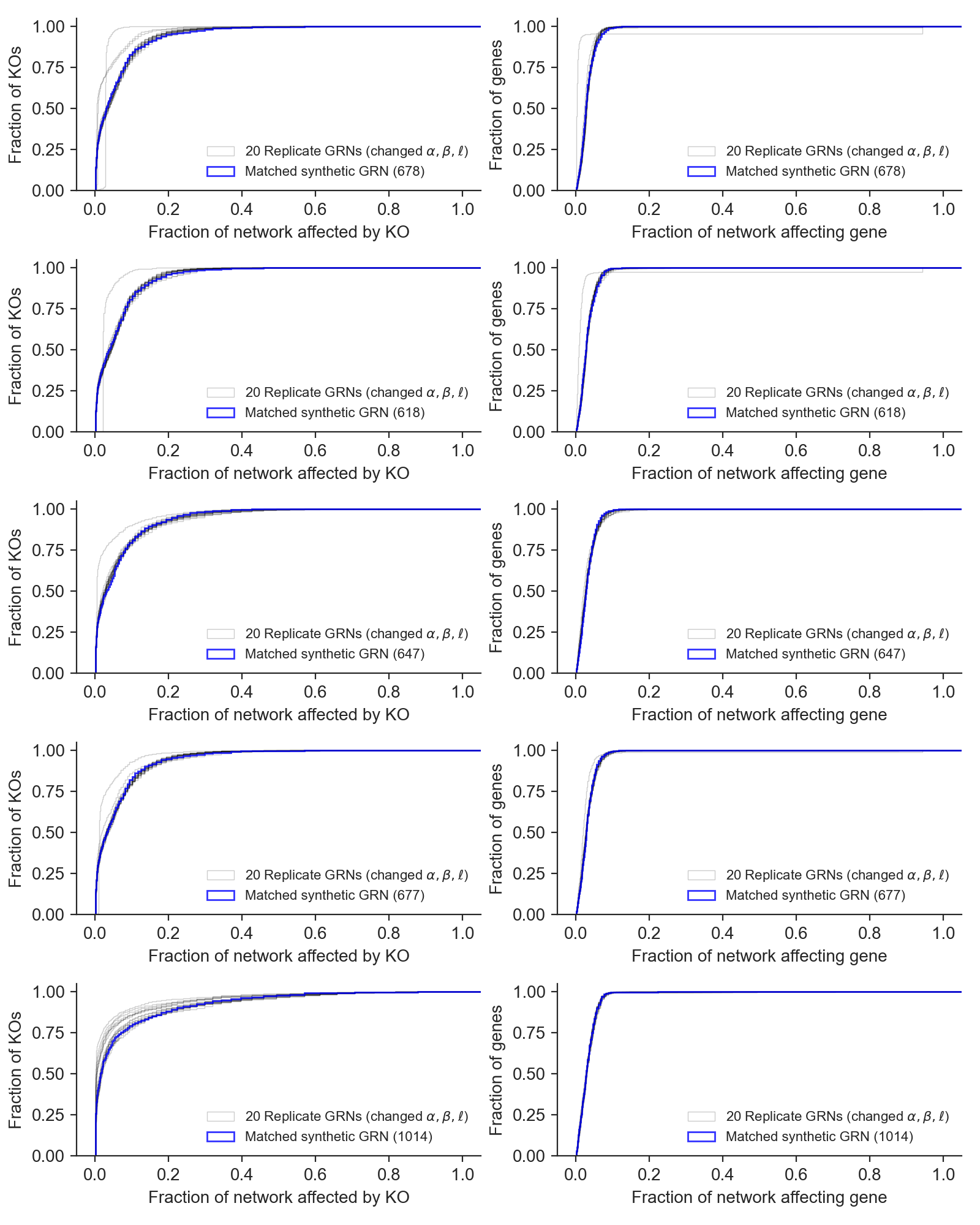

Supplement: S12 Fig — Replication of Fig 5A and 5B for the four GRNs closest to real Perturb-seq data. For each GRN, we resample the parameters of the expression model (α,ℓ,β; see Methods) 20 times and recompute the distribution of KO effects (outgoing, left panel; incoming, right column). The GRNs numbered 1, 2, 3, and 4 in Fig 5 are respectively numbered 647, 1014, 678, and 677 here. GRN number 647 (1) is the GRN used in Figs 6 and 7. (TIFF) [file pcbi.1013387.s015.tiff]

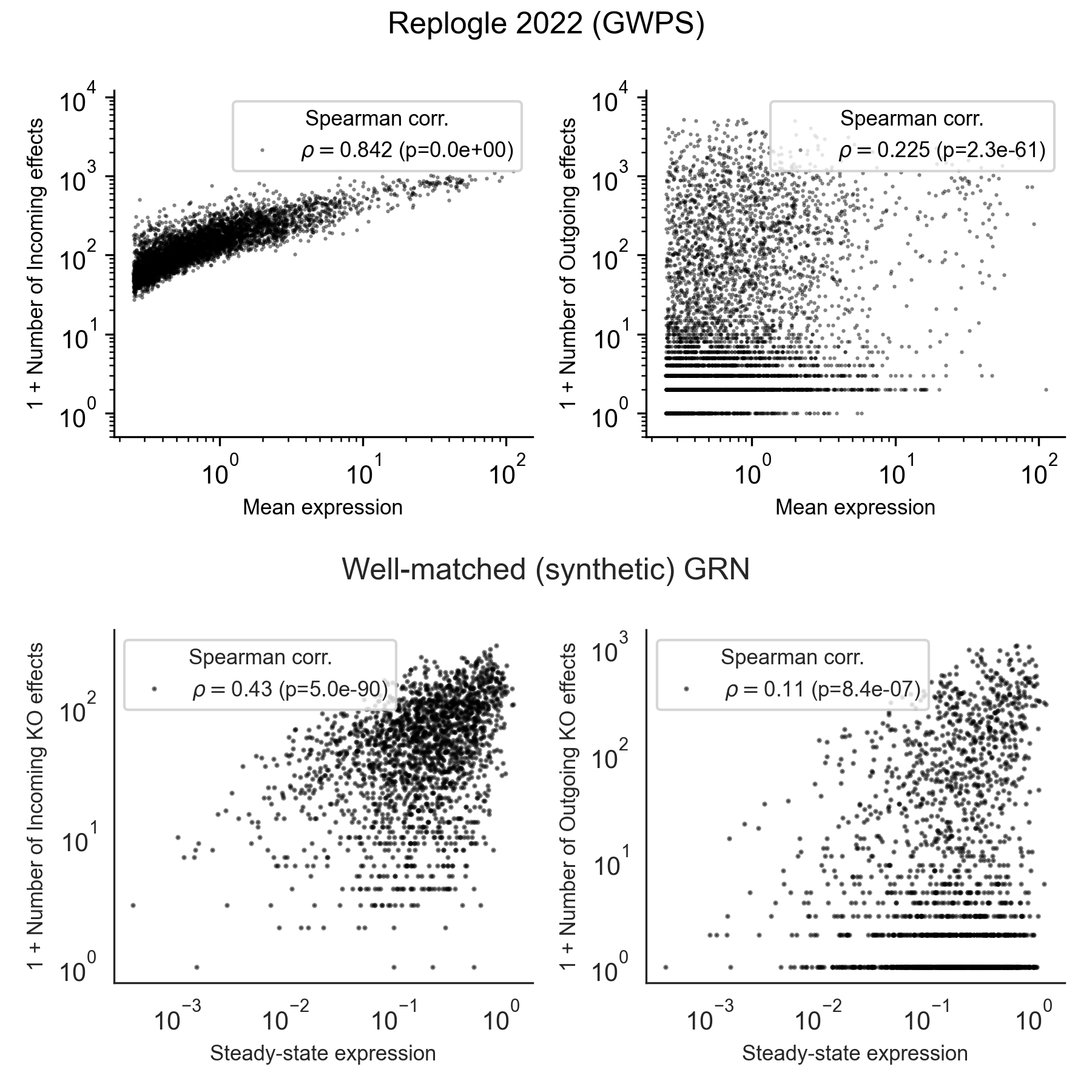

Supplement: S13 Fig — In the subsetted data from Replogle et al. [9], and in the focal GRN from Fig 6, we show the relationship between mean expression (in control cells in the experimental data; top panels) or steady-state expression (in the synthetic GRN; bottom panels) and the number of incoming (left) or outgoing (right) perturbation effects. Baseline expression relates to both of these quantities in both data sets: the relationships are stronger in the experimental data in part due to limits on detection power (especially important for incoming effects). (PNG) [file pcbi.1013387.s016.png]

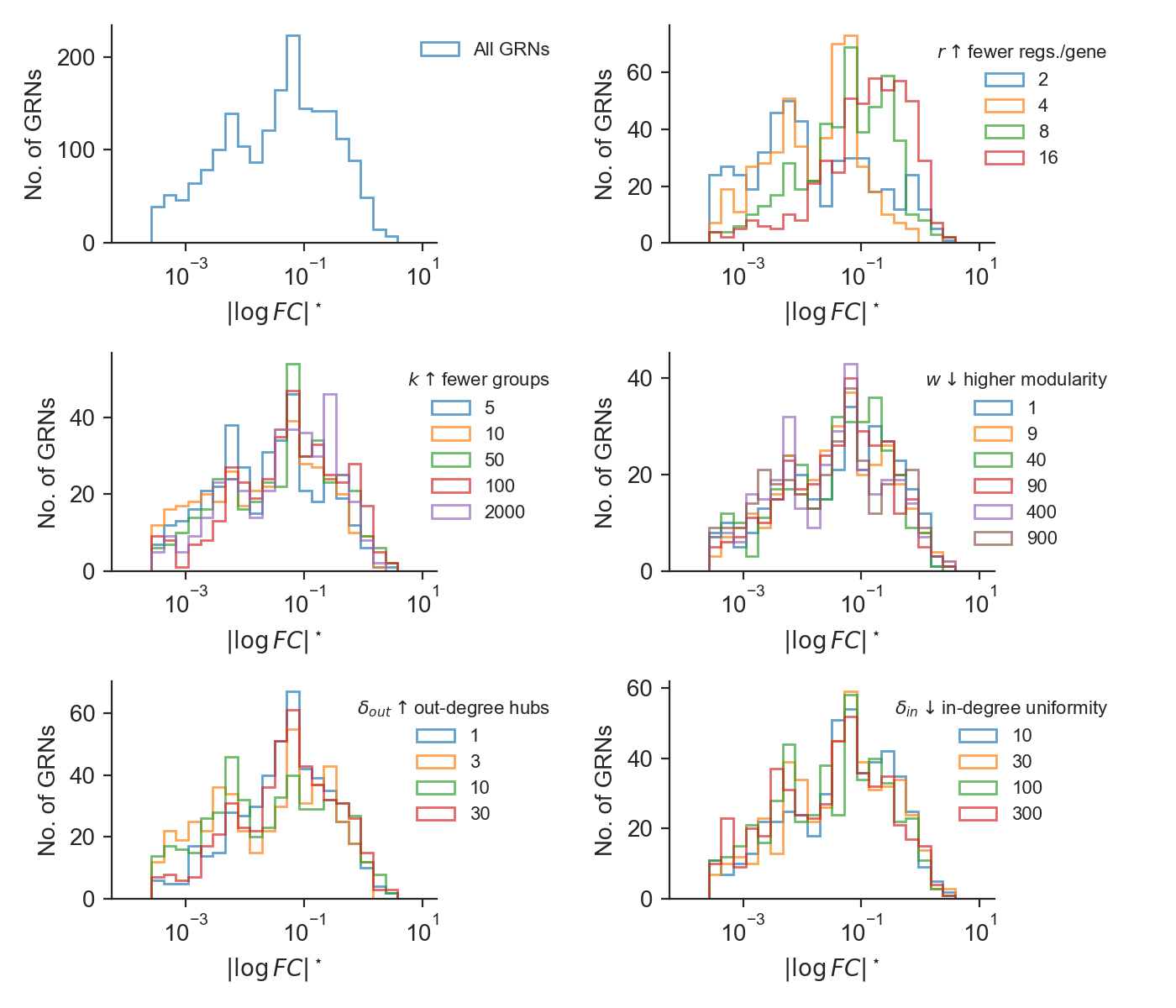

Supplement: S14 Fig — Critical values of |log2FC | used to match the discovery rate of real Perturb-seq data in Fig 5. The top left panel shows the distribution over all 1,920 GRNs; each other subpanel shows the conditional distributions for each generating parameter. We note a relationship between the number of regulators per gene (r), hub regulatory architecture (δout), and this critical value. (TIFF) [file pcbi.1013387.s017.tiff]

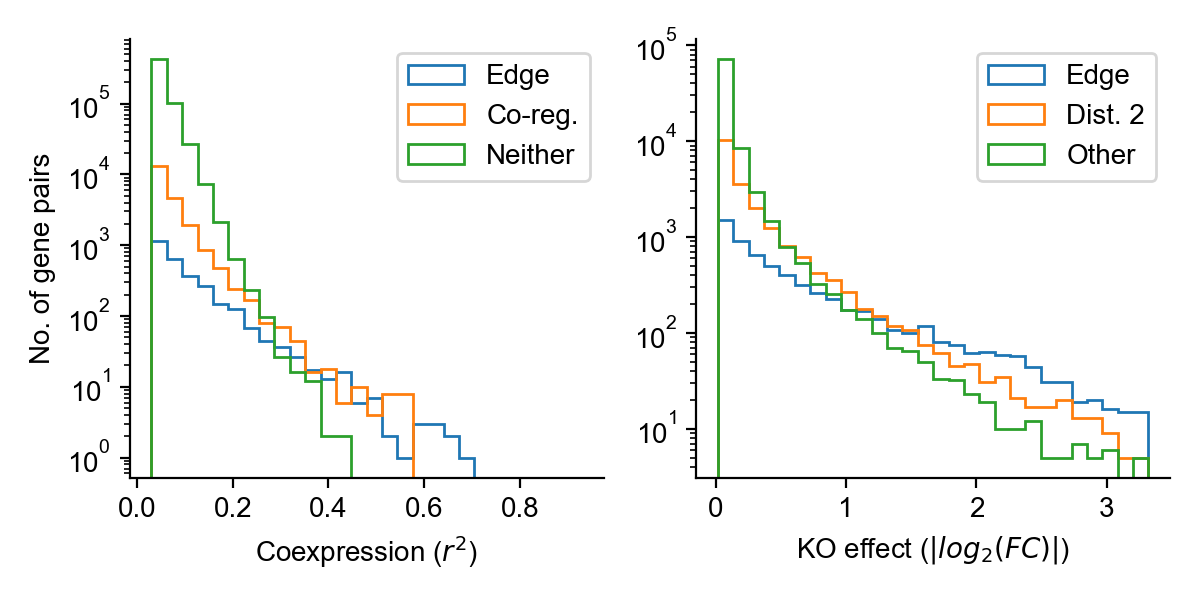

Supplement: S15 Fig — In the focal GRN from Fig 6, we show a histogram of coexpression values split by whether pairs of genes share an edge (“A regulates B, or B regulates A”, share a regulator (“A and B are coregulated”), or have another relationship (left panel). Similarly, for perturbation effects, we show the distribution split by whether pairs of genes share an edge (“A regulates B”), a path of distance 2 (“A indirectly regulates B”), or another relationship (right panel). At nearly all levels of coexpression, coregulation is more common than direct regulation. Meanwhile, direct regulation is more common than indirect regulation for the largest perturbation effects—note that the range of KO effects is clipped as in Fig 6. (TIFF) [file pcbi.1013387.s018.tiff]

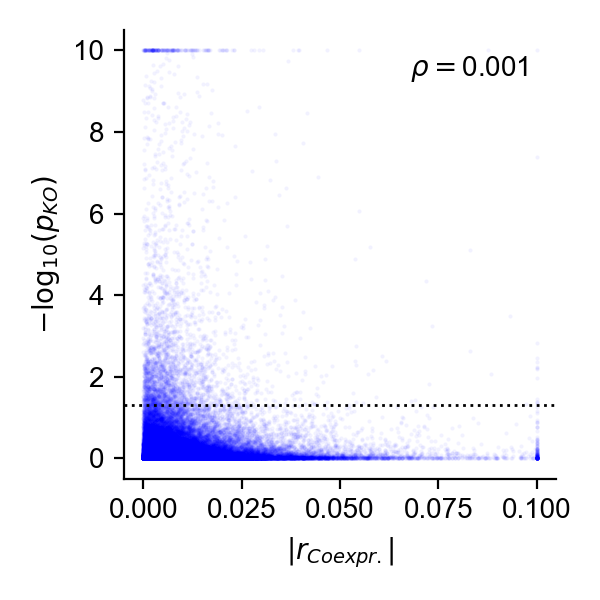

Supplement: S16 Fig — Same as Fig 6E, using data from our analysis subset of Replogle et al. 2022 [9]. Gene co-expression (x-axis) is the unsigned Pearson correlation between normalized single-cell gene expression data from unperturbed cells (clipped at |r|=0.1). Perturbation effects (y-axis) are pairwise log-transformed Anderson-Darling p-values for differences in gene expression distribution between perturbed and unperturbed states (clipped at −log10(p)=10). Rank correlation (Spearman’s ρ) is computed on the transformed but not clipped values of these two statistics. (TIFF) [file pcbi.1013387.s019.tiff]

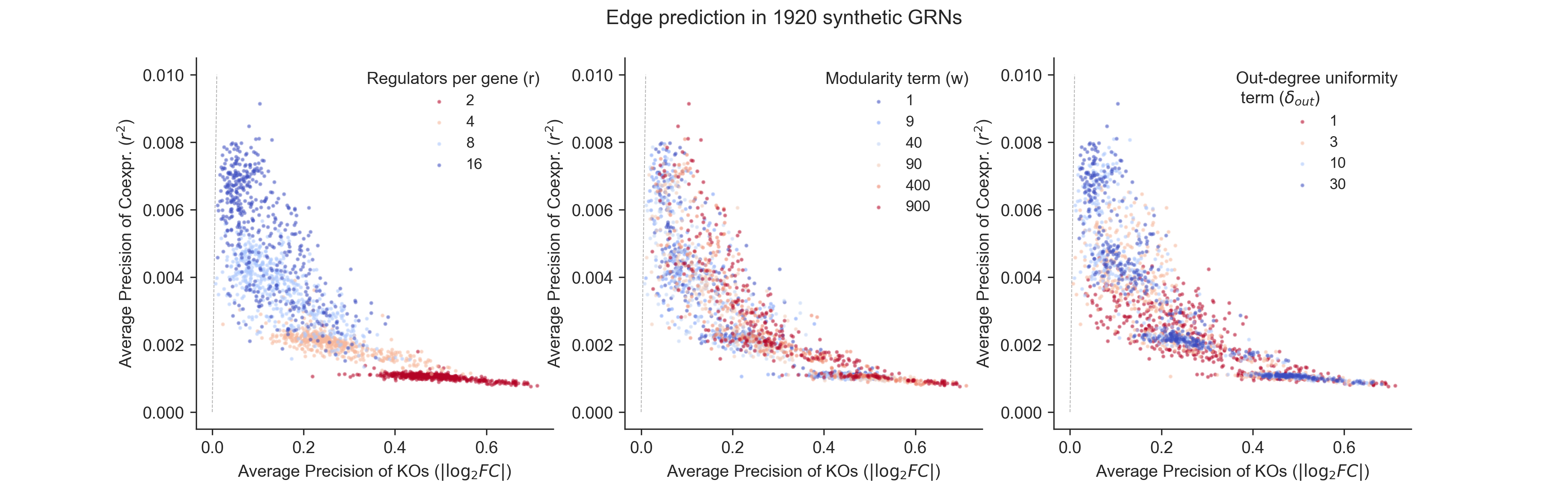

Supplement: S17 Fig — Performance of perturbation effects (x-axis, |log2FC |) and co-expression (y-axis, r2 between genes) in identifying edges. As a summary performance measure, we compute the average precision (AP) score during binary classification of pairs of genes as being connected an edge (ignoring direction). All 1,920 networks in the study are shown in both panels, and colored by parameters of interest: the sparsity parameter (r, left), the group affinity parameter (w, middle), and the out-degree parameter (δout, right). Across networks, sparsity, modularity, and degree uniformity degrade the performance of coexpression values, but enhance the performance of perturbation effects; but in every network, perturbation effects outperform coexpression (all points are below the dashed grey line, y = x). (TIFF) [file pcbi.1013387.s020.tiff]

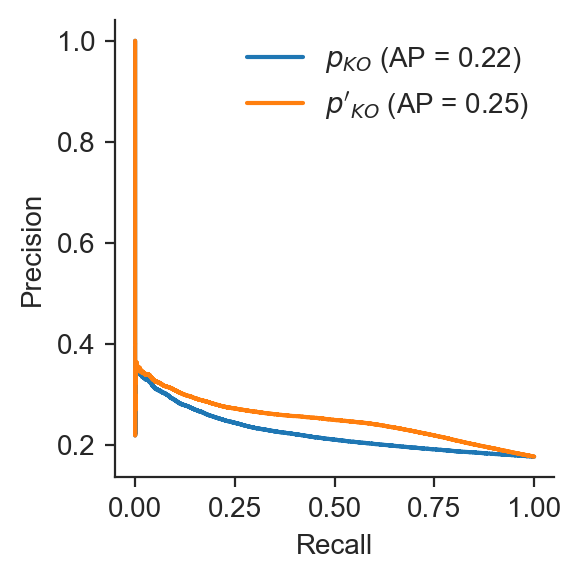

Supplement: S18 Fig — Performance of perturbation effect p-values (blue line, pKO) and re-weighted perturbation effect p-values (orange line, pKO′=pij/ni) in identifying pairs of genes with established protein-protein interactions, shared protein complex membership, or ChIP-seq links (Methods). In the prior expression, pij is the p-value for the response of gene j to a perturbation of gene i, and ni is the number of genome-wide significant effects (at FDR 0.05) that perturbation of gene i has on all other genes in the dataset. (TIFF) [file pcbi.1013387.s021.tiff]

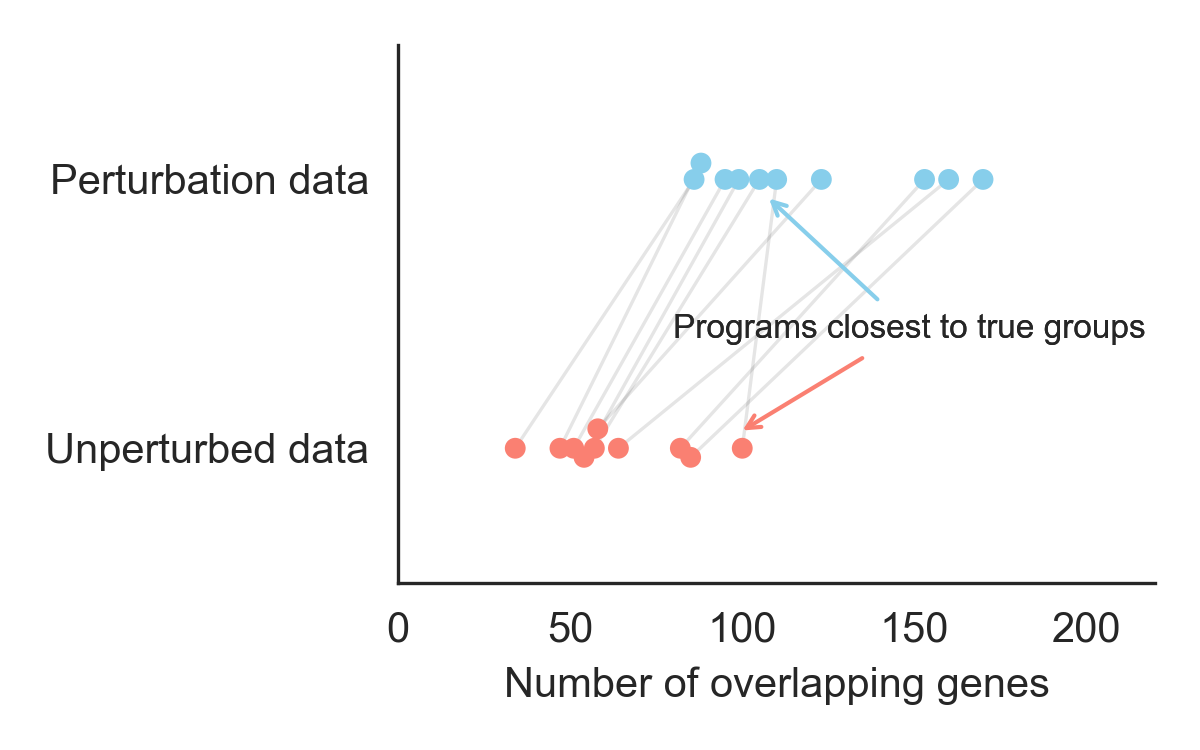

Supplement: S19 Fig — In the focal GRN from Fig 7, we show the overlap between each of the true groups (k = 10, shown as points in each of the bins on the y-axis) and its closest matching program (maximum overlap across all 50 gene sets, values shown on the x-axis). Points corresponding to the same true group are connected with a line spanning across y-axis bins. There is similar representation of all of the groups among the learned gene programs, regardless of input data type. (TIFF) [file pcbi.1013387.s022.tiff]

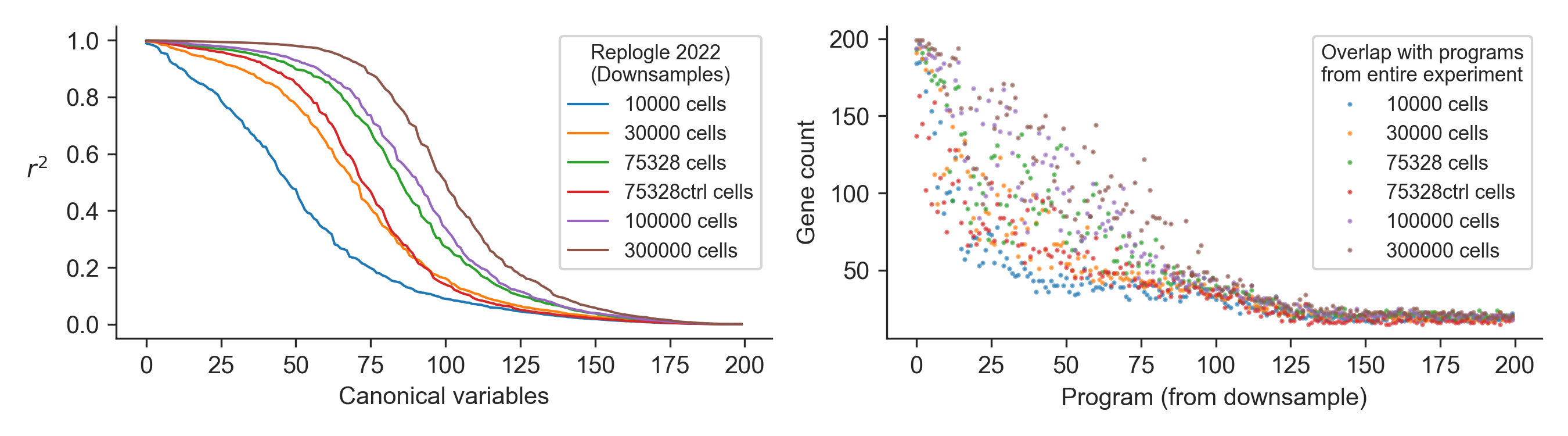

Supplement: S20 Fig — Same as Fig 7C and D—instead of taking downsamples of unperturbed cells from Replogle et al., 2022, we downsample the entire experiment to various study sizes. Here, the “entire experiment” is the normalized expression measurements of 5,247 genes in 932,593 control and intervened-upon cells which received one of the 5,247 perturbations in our analysis subset (Methods). We compare singular vectors (left) and programs (right) from the resulting downsamples of the entire experiment, as well as the subsets from Fig 7C and D. We note that the 75,328 control cells replicate the programs from the entire dataset comparably to 30,000 cells from the entire experiment. (TIFF) [file pcbi.1013387.s023.tiff]

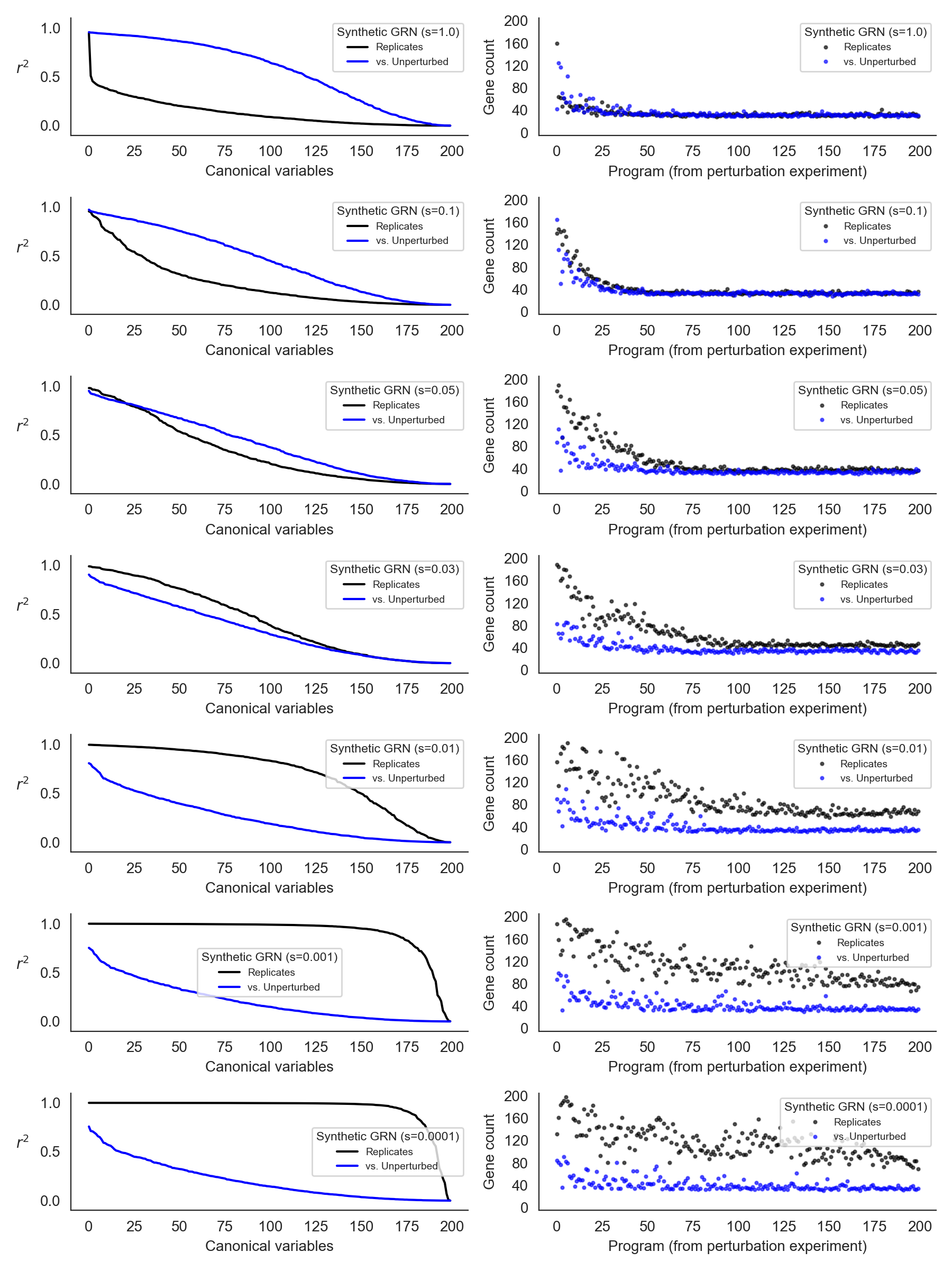

Supplement: S21 Fig — Same as Fig 7A and B for different levels of noise. We repeat CCA and analysis of gene programs as in Fig 7 (see Methods), varying the level of intrinsic noise (s). At low levels of noise (small s), replicates from perturbed conditions are much more similar to one another than to the unperturbed data. At high levels of noise (large s), the perturbed data are more similar by canonical correlation to the unperturbed data than to the replicate perturbed data; but programs derived from each of the singular vectors are equivalently reproducible across conditions. (TIFF) [file pcbi.1013387.s024.tiff]
