# Supplementary material for: Gene regulatory network structure informs the distribution of perturbation effects
Source: PLoS Comput Biol. 2025 Sep 2;21(9):e1013387. doi: 10.1371/journal.pcbi.1013387 (PMC12419648; doi:10.1371/journal.pcbi.1013387)
Supplement: Attachment — Submitted filename: grn-paper-review-response-20250127.pdf [file pcbi.1013387.s025.pdf]

Reviewer comments

Reviewer's Responses to Questions

Reviewer #1:

We thank the reviewer for their comments on our work. We would like to clarify that our paper proposes not only a method to simulate perturbations, but also to produce synthetic networks and model gene expression under a variety of conditions for these synthetic networks. The bulk of our analyses and results are focused on characterizing this model and evaluating it in the context of existing experimental data. We further offer analytical vignettes which highlight implications of our model for inferring regulatory network structures, and consider future applications of our techniques in the discussion. With this in mind, our responses to each of the reviewer's concerns are below.

The paper proposed a method to simulate perturbation on GRNs. My major concern:

1. There is a big gap between simulation and real-world application. Many details on the application on [9] is missing. It is hard to evaluation the method's performance in real-world applicaiton.

Our use of experimental data from *Replogle, et. al., 2022* (reference [9]) serves two purposes. First, in Fig. 1, we use simple summary statistics to motivate the design choices for our model. Later, in Fig. 5, we highlight that our extensions of prior network simulation techniques (specifically, of *Bollobas 2003*, reference [28]) provide improvements in describing properties of experimental data. Our intent is not to apply our model to the data from [9] to perform parametric inference (as stated in line 300 of the revised text):

> *"Although the focus of our work is not wholesale network inference, we do observe a coherent set of properties among the well-matched networks".*

Instead, we argue that the network underlying the data assayed in [9] exhibits sparsity, modularity, and degree dispersion to an extent that is captured reasonably well by a subset of the networks we simulated in the study. We further show (Figs. 7, S9, S11, and S14) that these GRNs recapitulate nontrivial similarities to experimental coexpression and perturbation data. We believe these results provide useful intuition for working with these data, and that this showcases the real-world utility and performance of our model.

2. The method sample many random GRNs, and simulated them and see their behavior. However, the results depend on how good the sampling is. But it is hard to know how many samples we need to have meaningful results. What if all GRN samples are not correct, then all the observations would be wrong.

We agree that it is not always obvious to determine the requisite sample size to draw robust conclusions from simulation studies such as ours. However, we have good reasons to believe that each of the claims in our paper are reasonably founded using the number of synthetic GRNs we simulated. We consider two categories of results:

First, with respect to network properties: as stated in line 495 of the revised text, we designed the experiment to sample parameter values which we believed completely span the biologically plausible range based on our prior knowledge:

"To cover these ranges, the values are spaced geometrically, and the extrema are chosen to overlap values which we believe are consistent with biological intuition for a network of n = 2,000 genes."

Within these ranges, we notice that the effect of any given parameter tends to be continuous across the values in instantiated networks (Fig. 4). Importantly, these trends tend to hold even when substantially varying the distribution of other network generating parameters (Figs. S5, S6, S8). This suggests that the network properties we describe are exerting independent influence on important summaries of the effects of perturbations.

Second, with respect to the implications of network properties for inference tasks: an important limitation in assessing the utility of different data sources for inference is a lack of high quality ground truth for real networks. We therefore compared our simulated data to summaries of the data from Replogle, *et. al.* [9] which are not limited by this absence of ground truth – specifically, we find that the synthetic networks exhibit meaningful similarities to the experimental relationships between perturbation effects and baseline gene expression (Fig. S9) and in baseline coexpression (Figs. 6E, S11). Moreover, the trends we observe are not limited to the exemplar network highlighted in the main text (e.g., the relative performance of gene co-expression and perturbation effects is similar in networks with similar properties – see Fig. S12).

3. How model parameters betas are determined. Are they also random? If so, then there are to many random effects in the model. All the conclusion could be different if we run the method again.

Regulatory effects in the gene expression model (*β_ji*) are randomly sampled, as in line 550 of the revised text:

"β_ji ~ (2 p_j − 1) · (1 + Half-Normal(0, 1)), under the assumptions that regulatory interactions have a minimum strength (|β_ji| ≥ 1). Here, p_j ~ Bernoulli(p = 0.8) is the probability that a regulator j acts as an activator rather than a repressor."

As mentioned throughout the text, we made modeling choices and assumed distributions of parameters that we argue constitute reasonable approximations to what may be biologically plausible. It is true that some of these assumptions will be violated in practice, and that there exist more faithful models of transcriptional activity which have different mathematical formulations (but are generally more computationally challenging to work with).

Since our generating algorithm is a novel model of biological networks, we spent the first section of our study characterizing the effects of the (network) parameters on simple, high-level summaries of the synthetic data. These parameters tend to exhibit independent and continuous effects on network susceptibility to perturbations (Fig. 4, S5), but it is also true that randomness in the expression model (e.g., variance in betas) contributes as well. We see this in our regressions on network parameters (summarized in Table S1, S2), in that there is modest residual variance in network susceptibility to perturbations. We now clarify this in the revised text, on line 268 (bold text is new):

In total, the network generating parameters explain just under half the variance in the fraction of the GRN which is either a hub KO (model r2 = 0.59) or hub target gene (model r2 = 0.46) – ***the remaining variance is attributable to randomness in the generating algorithm and in the expression model parameters*** *(Methods).*

However, we reiterate that the effects of the network parameters are strong enough to be detected even when averaging over variance from both the generating algorithm and in the expression model.

Furthermore, to the extent that our modeling assumptions are, indeed, assumptions, we focused the latter half of our work comparing our findings to experimental data. We found non-trivial similarities between GRNs simulated under our model and these data, with respect to both network properties (Fig. 5A-B) and to gene-level summaries (e.g., Fig. 6E, S11). Given the consistency of parameter effects under our model, and its similarities to experimental data in certain cases, we are confident that the overall findings of our work are robust to our modeling choices. However, we encourage future studies to consider the necessary and sufficient conditions to produce good models of complex systems like GRNs. We see our work as a key first step to better understand these properties (and their importance for experimental design and data analysis), and look forward to re-evaluating our assessments as our repositories of experimental data expand.

Reviewer #2:

The manuscript by Aguirre et al., “Gene regulatory network structure informs the distribution of perturbation effects,” introduces a method for generating gene regulatory networks (GRNs) that allows for the control of network properties through generation parameters. This is followed by a stochastic differential equations model to simulate perturbation effects on gene expression. The authors provide a thorough analysis of how network properties influence gene perturbation effects, demonstrating that synthetic networks generated by their method can recapitulate certain characteristics observed in experimental Perturb-seq data. Notably, the authors compare the utility of co-expression and perturbation data for GRN inference tasks, concluding that perturbation data is more effective for inferring regulatory edges, while both perturbed and unperturbed data identify similar gene programs.

The manuscript is well-written, easy to follow, and the dataset and code have the potential to be highly valuable for advancing the understanding and modeling of GRNs.

I have three comments that I believe could help clarify key points and enhance the study's impact.

We thank the reviewer for their comments and for their kind words about our manuscript. We hope our responses below are sufficient to address their concerns, and we appreciate their enthusiasm about the potential impact of our work.

1. Synergistic Regulation in GRNs
Synergistic regulation is a critical feature of biological GRNs. In the manuscript, the authors model the transcript synthesis rate as a sigmoid function of the combined linear effect of regulator expression. Could the authors clarify whether their model accounts for synergistic effects among regulators? If this aspect is not included in the current model, it would be important to address this limitation in the Discussion section.

Our model does not explicitly account for synergistic effects among regulators. Typically, a model of these types of effects would require the use of interaction terms (e.g., $x = \sigma(b_1x_1 + b_2x_2 + b_{12}x_1x_2)$). We found these models to be computationally demanding and difficult to parameterize from first principles, which motivated our choice of a linear model inside the sigmoid function. We have updated our discussion to clarify that cooperativity and antagonism were what we had meant when previously referring to “detailed models of biological synthesis”. Line 452 of the revised text now reads:

> *“For the sake of computational efficiency in quantifying expression for thousands of genes, we have also eschewed detailed models of the biological synthesis and experimental measurement of cellular RNAs (e.g., models that use the Hill equation or account for interactions between regulators)”*

However, we will further note here that the sigmoid function does introduce a kind of synergism and antagonism at the extremes of gene expression, since regulatory effects are buffered near zero and near a gene’s maximum expression value $1/\ell$. This essentially means there is antagonism between activators when their target gene is highly expressed and synergism when it is not; similarly, there is antagonism between repressors when their target gene is lowly expressed and synergism when it is highly expressed.

2. Bimodal Distribution of Hub Target Genes (Figure 4)
In the text for Figure 4A (Lines 220-221), the authors note, “However, in a subset of dense networks, most genes in the network are identified as hub target genes.” In fact, in other panels of Figure 4, the number of hub target genes often displays a similar bimodal distribution, independent of the network properties.

This is not observed for hub KO genes. Could the authors provide an explanation for this pattern? Are there unique characteristics in networks where nearly all genes are identified as hub target genes?

We believe this bimodality in the distribution of hub target genes is related to the stability of the gene expression steady-state of our model. As we state on line 232 of the revised text,

> *"However, in a subset of dense networks, most genes in the network are identified as hub target genes. This is related to the absence of stable equilibrium dynamics in the low-noise limit of the gene expression model (Fig. S4), which suggests that as GRNs become more dense and genes are subject to regulation by larger fractions of the network, the system is less likely to be stable."*

We assess this using a linear stability analysis of the gene expression stochastic differential equation in a low noise limit (see Methods section 4.2.2). There are a few reasons this instability could arise in a network: one is that regulatory cycles can introduce periodic oscillations in gene expression over time steps (which contributed to our choice to work with average measurements of gene expression over many simulated time steps). In general, instability means that the system is more likely to tend to a new equilibrium when perturbed. Indeed, these networks with unstable steady states were more likely to exhibit system-wide changes in expression in response to perturbation (Fig. S4).

We also believe that this bimodality is affected by binning. When summarizing the distribution of incoming and outgoing perturbation effects at the gene level, we binarized the distribution of perturbation effects first by size (|$\log_2$FC > 0.1|, to say an effect is large) and then by count (at least 100 large incoming or outgoing effects, to say a gene is a hub regulator or hub target). If we instead compute the number of large perturbation effects per gene in the entire network, we find a more unimodal distribution across generating parameters (Fig. S3).

In terms of network properties, we see that most genes are hub targets when the network gets sufficiently dense (Fig. S5, large *r*), and when the out-degree distribution is more Poisson-like (Fig. S6, large $\square_{out}$). In these networks, the typical gene has more direct regulators (more one-hop connections), and the regulatory architecture is more dispersed throughout the network (more two-hop connections, since there are fewer distal master regulators). When we look at a binarization of these incoming connections (as in Fig. 4), we get a bimodal distribution, even though the underlying data are continuous.

Finally, there is a simple reason why we see a binning effect for in-degree (hub targets) but not out-degree (hub regulators). When generating graphs, we enforce that all genes are subject to regulation, but not all genes in the network will end up being regulators (i.e., have any outgoing connections at all). This means that in more dense (or dispersed) networks, any gene could end up a hub target, while some genes in these same networks will not even be regulators at all.

3. Data and Code Availability
I strongly recommend that the authors make their code and synthetic network data publicly available, including proper references to these resources in the manuscript. While the authors state in Lines 67-68 that their tools are available on GitHub, no URL is provided. Additionally, the "Data and Code Availability" section indicates, "All relevant data are within the manuscript and its Supporting Information files." However, I was unable to locate the synthetic network data (network structure and simulated expression) in Supporting information files. As the authors noted in the Discussion, their technique could significantly benefit model development and benchmarking. Making these datasets publicly available would enable further studies of GRN properties and inference, greatly enhancing the utility and impact of this work.

We thank the reviewer for calling our attention to this oversight. We have updated the github links in the text, and have confirmed that the repository is publicly available.

Reviewer #3:

In this manuscript, the authors present a novel analysis of perturb-seq data through the lens of gene regulatory networks. By comparing real-world perturb-seq data to synthetic perturbation data, they offer a fresh perspective on interpreting noisy single-cell data. The manuscript is well-structured and clearly written. I recommend to accept this manuscript for publication.

We thank the reviewer for their kind words.
