# Supplementary material for: Gene regulatory network structure informs the distribution of perturbation effects
Source: PLoS Comput Biol. 2025 Sep 2;21(9):e1013387. doi: 10.1371/journal.pcbi.1013387 (PMC12419648; doi:10.1371/journal.pcbi.1013387)
Supplement: Attachment — Submitted filename: ko-paper_second-review-response_20250619.pdf [file pcbi.1013387.s026.pdf]

Letter from the Editors

Dear Dr. Aguirre,

Thank you for submitting your manuscript to PLOS Computational Biology. After careful consideration and in-depth discussion among the editors, we feel that while it has merit it does not fully meet PLOS Computational Biology's publication criteria as it currently stands. In addition to unsatisfactory response to reviewer 1, the editors have additional concerns. We invite you to submit a revised version of the manuscript that addresses the points raised during the review process.

However, we understand that the paper has been under review for a long period of time. If the authors choose to not address these concerns, the paper will be rejected with the option of transfer to PLOS one. We note that final acceptance is still subject to authors fully addressing the reviewer and editor comments. We understand this is a non-trivial ask and therefore we offer the transfer option. The editors are below.

Please submit your revised manuscript within 60 days **Jun 18 2025 11:59PM**. If you will need more time than this to complete your revisions, please reply to this message or contact the journal office at ploscompbiol@plos.org. When you're ready to submit your revision, log on to https://www.editorialmanager.com/pcompbiol/ and select the 'Submissions Needing Revision' folder to locate your manuscript file.

Please include the following items when submitting your revised manuscript:

- ☑ A rebuttal letter that responds to each point raised by the editor and reviewer(s). You should upload this letter as a separate file labeled 'Response to Reviewers'. This file does not need to include responses to formatting updates and technical items listed in the 'Journal Requirements' section below.
- ☑ A marked-up copy of your manuscript that highlights changes made to the original version. You should upload this as a separate file labeled 'Revised Manuscript with Track Changes'.
- ☑ An unmarked version of your revised paper without tracked changes. You should upload this as a separate file labeled 'Manuscript'.

If you would like to make changes to your financial disclosure, competing interests statement, or data availability statement, please make these updates within the submission form at the time of resubmission. Guidelines for resubmitting your figure files are available below the reviewer comments at the end of this letter

We look forward to receiving your revised manuscript.

Kind regards,
Emily Miraldi, Ph.D.
Guest Editor
PLOS Computational Biology

Sushmita Roy, Ph.D.
Section Editor
PLOS Computational Biology

Shihua Zhang
Section Editor
PLOS Computational Biology

Additional Editor Comments:

In mammalian systems, ground truth knowledge of GRNs is lacking, and generating synthetic networks to explore the relationship between network parameters and experimentally measurable GRN outputs (e.g., gene expression responses to systematic gene KO) is a valuable contribution and will be of interest to the computational biology community. However, there are opportunities to improve the rigor and communication of the work. Please see below.

We thank the Editors and Reviewer #1 for the additional comments. We have expanded our Introduction and Discussion sections to further motivate our work in light of prior studies on network inference and current challenges in the field. We also discuss limitations and extensions of our work for other biological modalities (e.g., protein interactions) models of gene expression (e.g., molecular kinematics).

We have also added several additional analyses (with corresponding Supplementary Figures) towards enhancing the rigor of our study and the robustness of our results. Specifically, we have conducted sensitivity analyses with respect to the hyperparameters of our expression model and our procedure to match data from synthetic GRNs to experimental data. We have also added a concrete example of how to make use of our results to improve network inference on real data, and we have added several descriptive analyses and figures that clarify details of our graph simulation algorithm, expression model, and matching procedure.

We believe that these additions have considerably improved the quality and communication of our work, and hope that our manuscript will be suitable for publication in PLoS Computational Biology. We thank you again for your time and consideration.

Major Concerns:

1. The DREAM literature showed that GRNs inference methods performing well on synthetic GRNs have failed to translate that performance to real datasets.

We agree that this has been a key finding of the DREAM challenges, which we now cite as additional motivation for our work. We suspect that one reason for the discrepancy in performance on real and simulated data is a mismatch between topological properties of the causal networks used for benchmarking and of the unknown networks assayed experimentally. We have added additional background on this (and related crowdsourced inference challenges) in the introduction:

> "Meanwhile, empirical competition-based benchmarking studies of GRN inference have revealed several challenges in the application of network inference approaches to real experimental data. In these competitions, simple heuristic approaches often perform best, as do techniques that leverage perturbation data, assume network sparsity, or ensemble multiple predictions. Complexity in regulatory network topology and in gene regulation as a biological process remains a key obstacle to refining inference approaches and establishing reliable benchmarks. Pernicious gaps persist between model performance when using synthetic networks for evaluations and performance when using silver-standard ground truth experimental data."

2. To demonstrate impact, authors would need to show how leveraging insights from their synthetic GRN analysis led to improvements to a GRN inference method that could then be evaluated using standard performance criteria (precision-recall of a gold standard network in K562, e.g., leveraging leave-out form the CRISPR screen, rich TF ChIP-seq from ENCODE...)? However, this is a nontrivial ask and beyond the scope of a revision (or the goals of the study as argued in the rebuttal).

Our work suggests several ways to improve the construction and benchmarking of GRN inference techniques. Indeed, our results on the key properties of GRNs can be incorporated into many different GRN inference approaches. To provide a concrete example of the practical use of our findings for inference, we have added an analysis inspired by the recent CausalBench inference challenge (refs. 10

and 11 in the text). In Figure S18, we show that the p-value ranks for genome-wide significant perturbation effects from the Replogle et. al. dataset can be used to identify pairs of genes that are linked by protein-protein and ChIP-Seq interactions. One of our main theoretical findings is that network activity in real GRNs tends to concentrate in regulatory hubs (i.e., nodes with large out-degree). This suggests that biasing edges to favor regulatory hubs should improve the quality of edge inference. Indeed, ranking by p-value can be improved by dividing each p-value by the number of genome-wide significant perturbation effects which originate from the perturbed gene (i.e., ranking on p_{ij}/n_i, where p_{ij} is the p-value for the effect of perturbing gene i on gene j and n_i is the number of genes that respond to perturbing gene i). The intuition is that this modification slightly lowers the inclusion threshold for outgoing edges from putative master regulators. Even this simple approach to incorporating prior knowledge from our theoretical results results in an increase in precision. Based on this simple analysis, we believe that incorporating our generative model as a prior (or even just using our general insights about the properties of GRNs) in more complex inference methods, will be similarly beneficial.

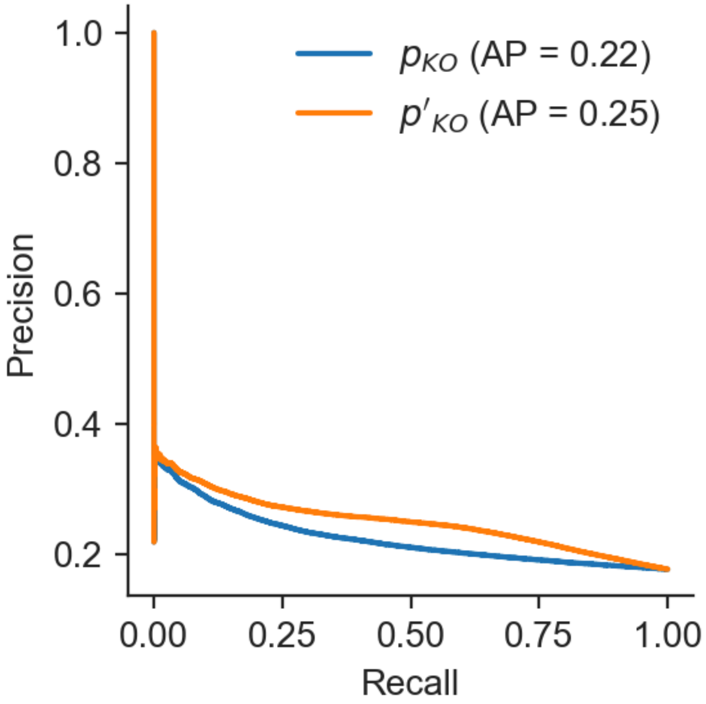

We make note of this finding in Results (Section 2.7.1: “Discovering pairwise interactions”), as below:

> “In particular, we anticipate that incorporating prior information on structural properties of regulatory networks will improve GRN inference techniques. As an example, we show that biasing the rank ordering of perturbation effect p-values from the Replogle et. al. dataset (to encourage edges to be drawn out from putative master regulators) improves correspondence with pairwise gene links from external sources on protein-protein and ChIP-seq interactions (**Methods**; **Fig. S18**).”

3. There are a variety of GRN definitions in the literature. Please clearly define that your GRN allows regulatory interactions between any genes in the network. In contrast, another GRN definition limit to regulatory interactions from protein TFs to genes. You are clearly not using this TF-centric GRN definition, as your network generating algorithm allows for regulatory interactions between all genes (direct or indirect) and this makes sense given eventual goal to compare to the K562 screen. However, there are a couple of places in the text where you muddy the water by invoking “TFs”. Please replace “TFs” with “regulators” in Fig. 2B, for example. More broadly, it will be valuable to discuss limitations of your GRN definition in Discussion, as your ODE model assumes that the

transcript of any gene can regulate transcription of any other gene, when biologically a gene regulatory network involves: mRNA → protein → protein-protein interactions → TFs → gene expression. This is a limitation of your GRN network model and it would be of value to the readership to discuss. (Relatedly, how might this more complex biological process be embedded into your beta coefficients or addressed experimentally and/or computationally?)

We thank the editors for the opportunity to clarify our conceptualization of GRNs. Our definition has two components: a regulatory network and an expression model. It will be helpful to consider these two components separately when discussing their strengths and limitations.

Here, we define the regulatory network in the causal sense – namely, an edge from gene A to gene B in the network means that the expression of gene A results in a direct causal effect on the expression of gene B. Beyond this assumption of causality, our model is deliberately general with respect to the mechanism by which regulation occurs. We have therefore replaced "TFs" with "regulators" in Fig. 2B and made analogous edits throughout the manuscript to use "regulator" in place of "transcription factor". We have further clarified that there is no restriction in the network generating algorithm which prevents any gene from acting as a regulator, and that there is likewise no forced partition of the network into "regulators" and "non-regulators" (though the causality assumption does specify a "regulator" and "target" for each edge). We have updated the text with the following:

> "We note that this algorithm does not pre-specify a partitioning of genes into regulators (with an outgoing edge) and non-regulators (without an outgoing edge); as formulated, any node can receive an outgoing edge, and so any gene in the network can act as a regulator."

Meanwhile, our gene expression model draws inspiration from models of transcriptional synthesis, but we chose the specific functional form of the model as a compromise between a simple linear model (most common in the causal inference setting) and more complex non-linear ODE models (more commonly used in chemical or systems biology). This compromise also enables us to specify a small number of hyperparameters for the expression model while also enforcing the biological assumptions that gene expression is non-negative and cannot be driven to arbitrarily high levels. This formulation also enables convenient computation: it would be comparatively much more challenging to sample parameters and perform simulations from a model with kinematic terms (e.g., from the Hill equation), higher-order binding logic (e.g., regulatory interactions with Boolean logic), or multi-step processes of biochemical synthesis (e.g., a fully specified model of splicing or translation). We acknowledge these simplifying assumptions in the Discussion section and caution that there are several settings in which more detail is warranted. We have expanded this discussion as follows:

> "Here, we have highlighted the utility of our approach with simulations to develop intuition about key properties of GRNs, particularly in the context of molecular perturbations and coexpression data. While our study design draws inspiration from recent works using Perturb-seq, we also acknowledge that our model makes simplifying assumptions. In focusing on the equilibrium dynamics of cells of one type, we have ignored developmental trajectories and cell-type heterogeneity within tissues, both of which modify our assumptions about regulatory network structure. For the sake of computational efficiency in quantifying the expression of thousands of genes, we have also eschewed detailed models of the biological synthesis and experimental measurement of cellular RNAs (e.g., models that use the Hill equation or account for interactions between regulators). For the same reason, our model also does not account for post-transcriptional activity (e.g., models of translation and protein-DNA and protein-protein kinematics), although the regulatory parameters of our expression model (i.e., beta coefficients within the sigmoid) may have some approximate relationship with parameters in a more fully specified biophysical model of expression regulation.
>
> "In future work where it is critical to model protein kinematics or to match distributions of RNA count data from experiments, we encourage modeling these complexities. We note that as in prior work, such models of protein translation, RNA observation, or other kinematic activity could be layered on top of our baseline expression model to produce data with more desirable

summary statistics. Moreover, our modeling approach is flexible enough to accommodate an entirely separate expression model which could be specified given an underlying causal graph. To facilitate this type of extension to our model, we have provided our analysis code on github, with separate implementations of our graph generating algorithm and expression model."

4. Fig. 5C shows properties of the 100 synthetic GRNs matching the KO perturbation distributions in the K562 screen. This is a key result. How were K-S statistics on 5A and 5B distributions combined (average rank)? Was there a natural break point in the combined similarity scores that would be more appropriate than 100? How robust are the results to the top 100 cutoff? What if you look at the top 5-10 most similar synthetic GRNs, do conclusions change? One could easily add data points of different colors corresponding to top 1, top 5, top 10, top 50 on 5C… Could be helpful to model similarity of synthetic networks' to K562 distributions as a function of synthetic network parameters, to further pinpoint those parameters most impacting similarity – but likely beyond the scope of this manuscript.

Our main claim in Figure 5C is that real data exhibit patterns which are *more consistent* with networks generated under assumptions of sparsity, modularity, and a heavy-tailed out- but not in-degree distribution. While we leave estimating the best-fit generating parameters for particular datasets as an exercise for future study, we agree that we can further assess the extent to which this consistency holds among our sample of simulated GRNs. We have performed the suggested sensitivity analysis and added it as a Supplementary Figure (S11), which we have included below:

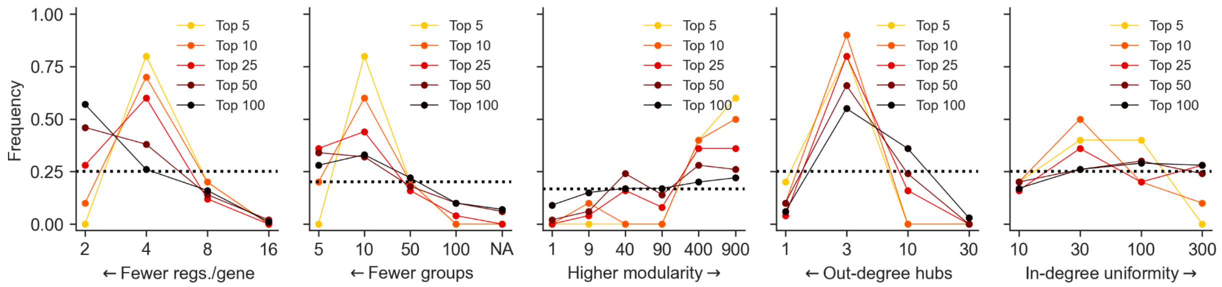

We have made an additional note of this result in Section 2.6 of the Results ("Comparing with experimental data"):

"Moreover, these results are also consistent across rank-based thresholds for the matching (**Fig. S11**) and across resampled parameters for the expression model, given the same causal network underlying the GRN (**Fig. S12**)."

5. The parameterization of the stochastic ODEs is random. A major goal of the study is to connect the topology-driving parameters used in network topology generation to simulations from the stochastic ODEs. Across random stochastic ODE parameterizations for the same network topology, how reproducible are the KO perturbations and resulting CDFs of % genes perturbed per KO and % KOs effecting genes (e.g., Fig. 5A, B) across random stochastic ODE parameterizations? To relate network topology parameters to KO simulations, it is important to establish that the CDFs are robust to random parameter sets. (Or to average across random parameterizations, if the KO simulations are sensitive to the random parameterization.) This is something that could be examined, say, with 5 or so random parameterizations of the 4 synthetic networks whose KO distributions were most similar to the K562 screen results.

We agree that it is worth considering the effects of randomness in the expression parameters, and have performed the suggested re-simulation analysis of these GRNs. The results of the new analysis are included in a new Supplementary Figure (S12), which is reproduced below.

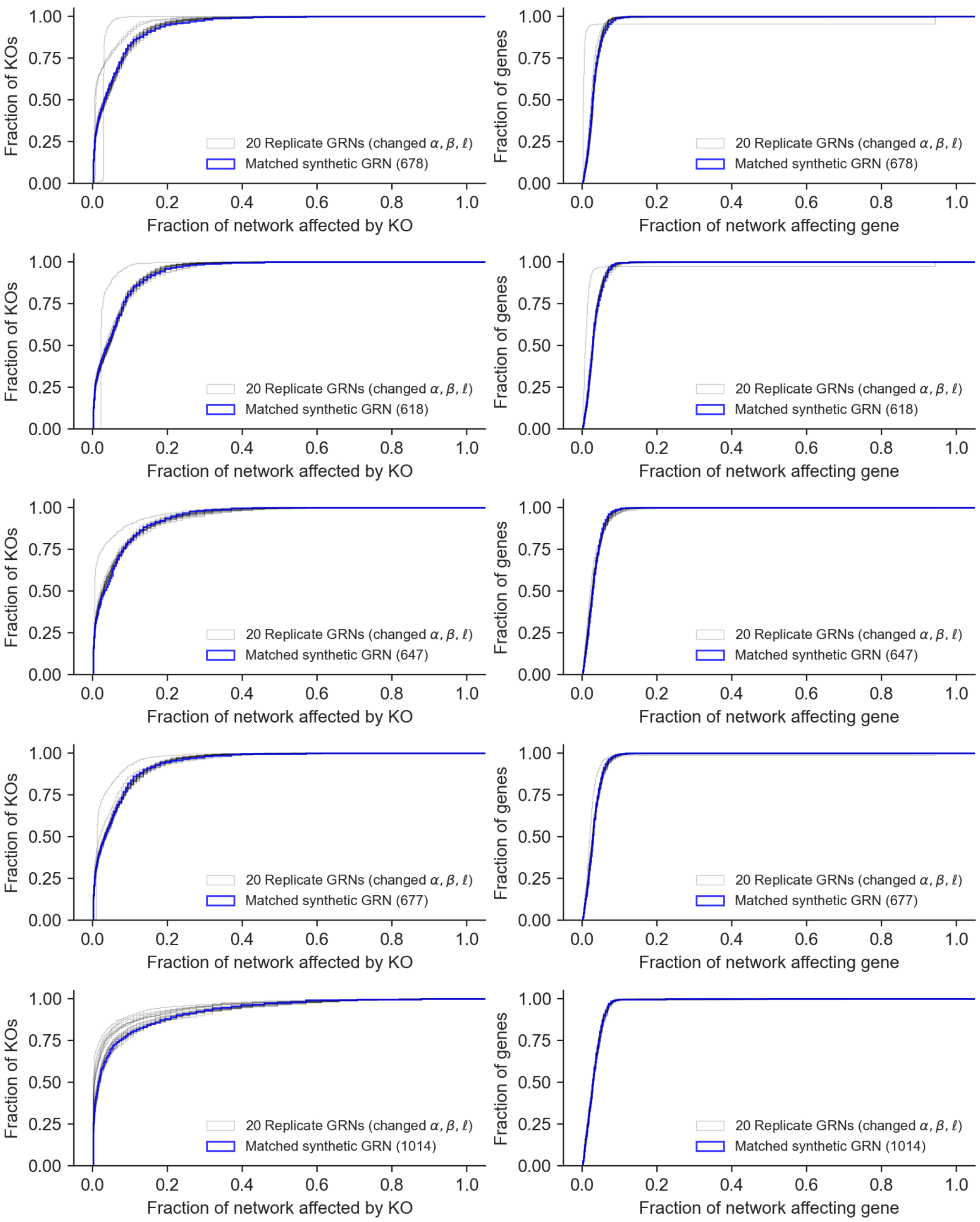

We note this result in Section 2.6 of the Results ("Comparing with experimental data"):

> "Moreover, these results are also consistent across rank-based thresholds for the matching (**Fig. S11**) and across resampled parameters for the expression model, given the same causal network underlying the GRN (**Fig. S12**)."

Beyond this new analysis, we have further reason to doubt that randomness in the expression model is the key driver of variance in these CDFs across networks: if this were the case, we would have had null results in the regression analysis in Fig. 4 (where network properties explain a majority of the variance in

two key quantiles of the distribution), and in the matching exercise in Fig. 5 (where there is enrichment of networks close to the Replogle et. al. study in key areas of our network parameter space).

Minor concerns:

1. In section 2.5, paragraph 1, genes whose equilibrium expression was below 10^-4 were removed from GRNs, but later (Fig. 5) the major network stats reported are based on the fraction of genes impacted by KO. How did that effect the denominator in Fig. 5A, B fractions? E.g., are you looking at only 600 genes or closer to 2000? Would be helpful to show a distribution of % genes above noise if they were a significant fraction of the 2000 total.

In all networks, the vast majority of genes are above the noise threshold and are included in analysis. We have plotted this distribution in a new Supplementary Figure (S9), which we show below:

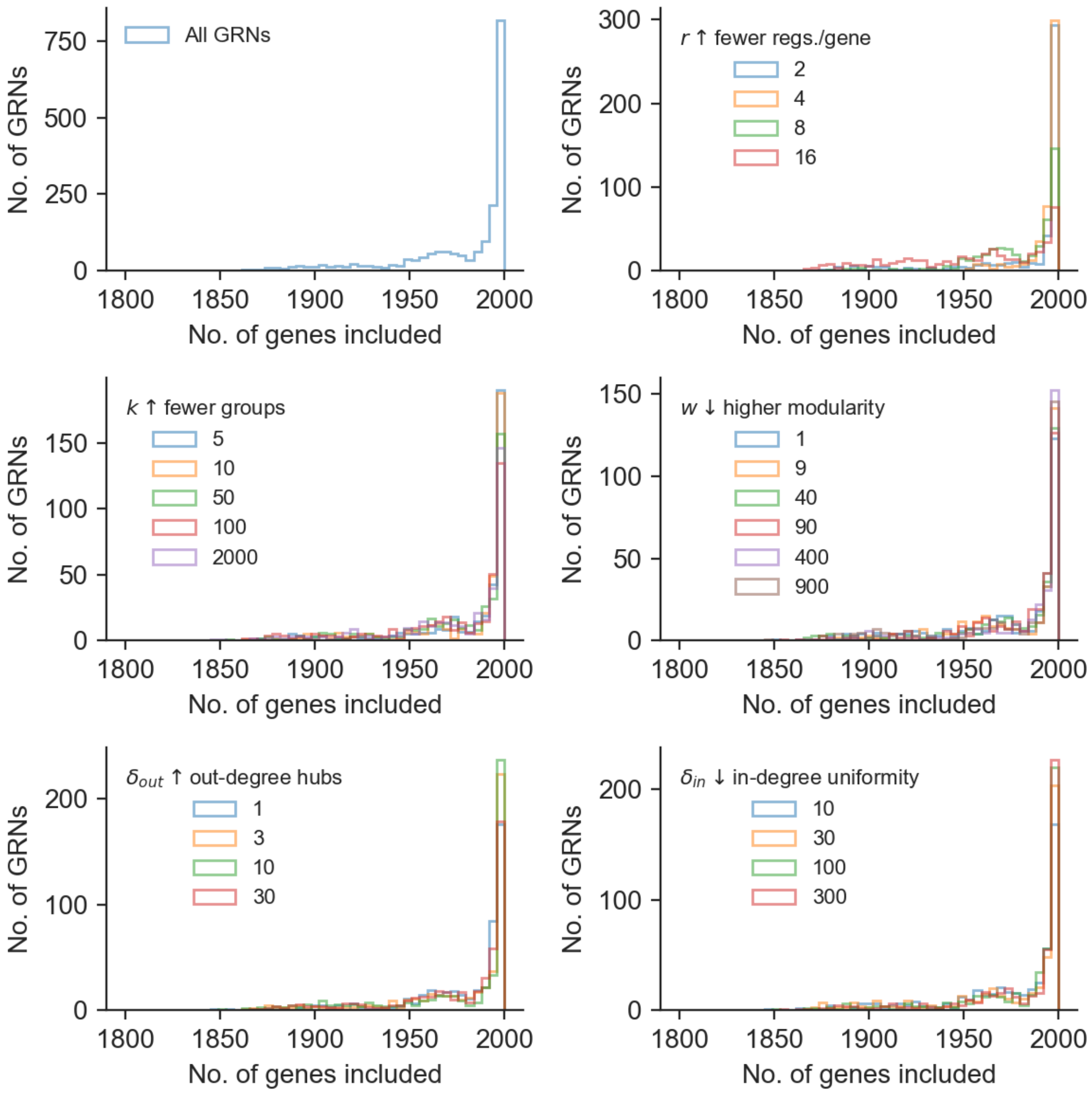

2. In section 2.6 / Fig. 5, to match perturb-seq data, genes were thresholded at the top 3% of absolute log2-FCs. On average, what log2-FC value did this correspond to? (10% (similar to cutoff used in earlier figures), something much smaller 10^-3?) Would be helpful to show the distribution FC cutoffs corresponding to 3%, to give readers a sense of effect size in model.

This threshold corresponded to a median log2 fold-change of 0.05 across networks, though there is substantial variance in this quantity (which is expected due to the analogous result shown across percentiles in Fig. 3C). We agree that these data offer a helpful insight into the behavior of our model, though at the same time we caution against interpreting these fold-changes on equal footing with the Anderson-Darling *p*-values from Replogle et. al, as the effects of biological, experimental, and sampling noise are not known *a priori*. We now show the distribution of this thresholded effect size in Supplementary Figure (S14), which is below:

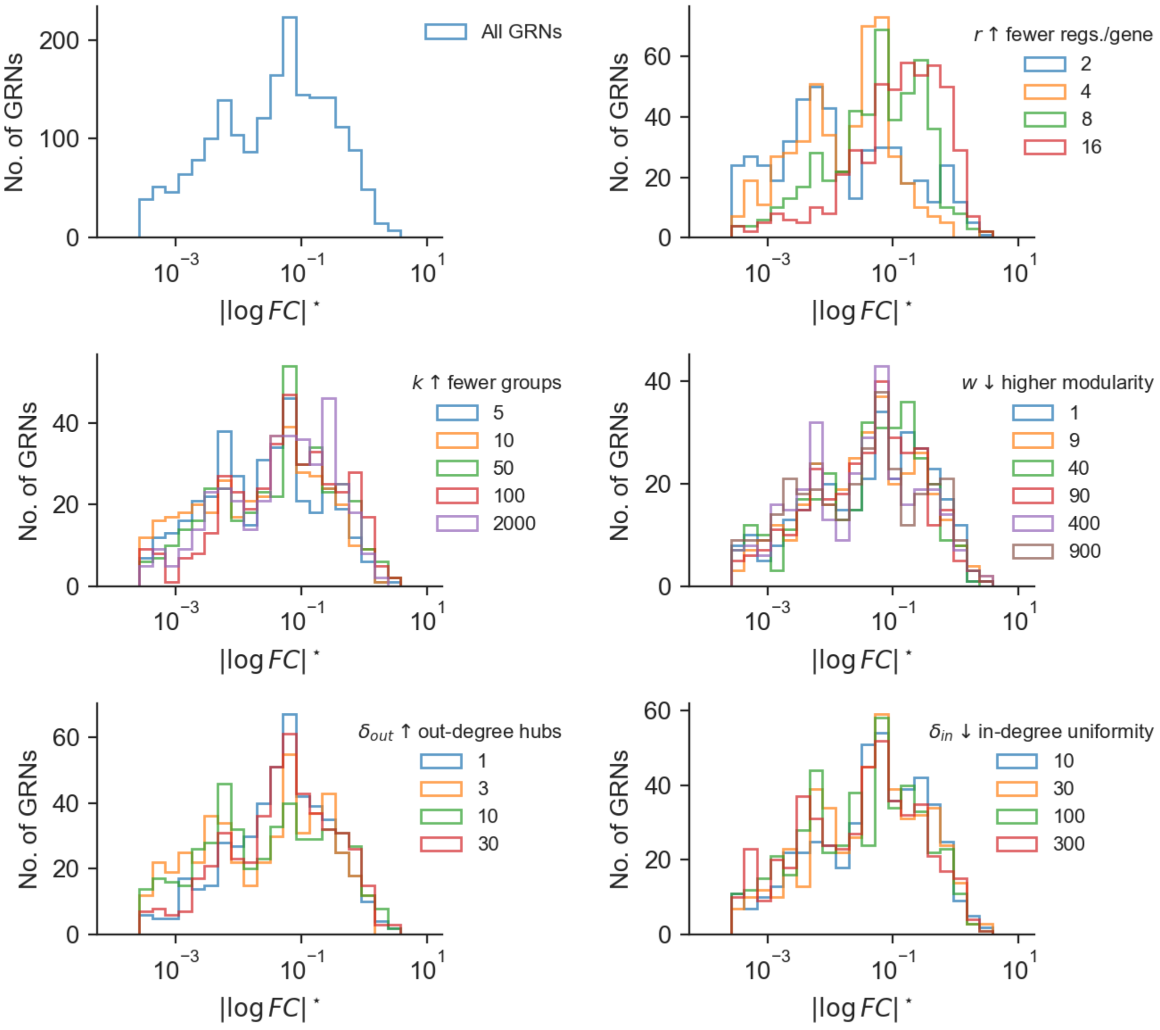

3. Fig. 1 – add y axis quantification to 1C

We have added this quantification to the figure, as below.

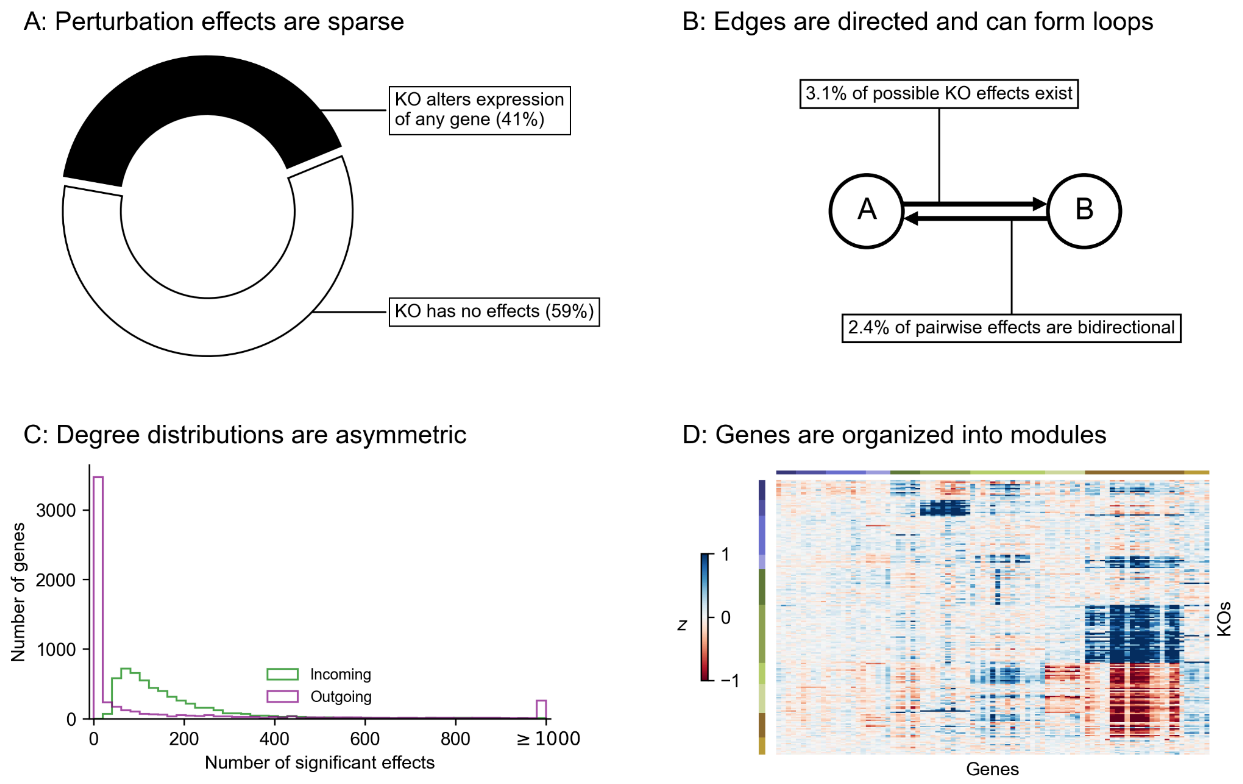

4. Fig. 2B – It's hard to get a visceral sense for the out-degree CV, would be beneficial to show some out-degree distributions for GRNs at particular delta_out values. For example, for out-degree CV = 5. What max out-degree does that correspond to? (200 or 2000 targets per TF?) Or better yet, would be helpful to show distributions of the max and mode out or in degrees for delta_out and delta_in parameters. Also, please improve the figure legend so that abbreviations (e.g., CV) are defined in legend, in addition to text.

We have clarified the abbreviation "CV" in the Figure 2 legend, as below:

> "Note that in the left two panels, CV is the coefficient of variation of the in- or out-degree distribution, i.e. the mean degree over its standard deviation."

We have added a new Supplementary Figure (S1) that shows how the in- and out-degree distributions change as a function of the delta parameters. The figure is below.

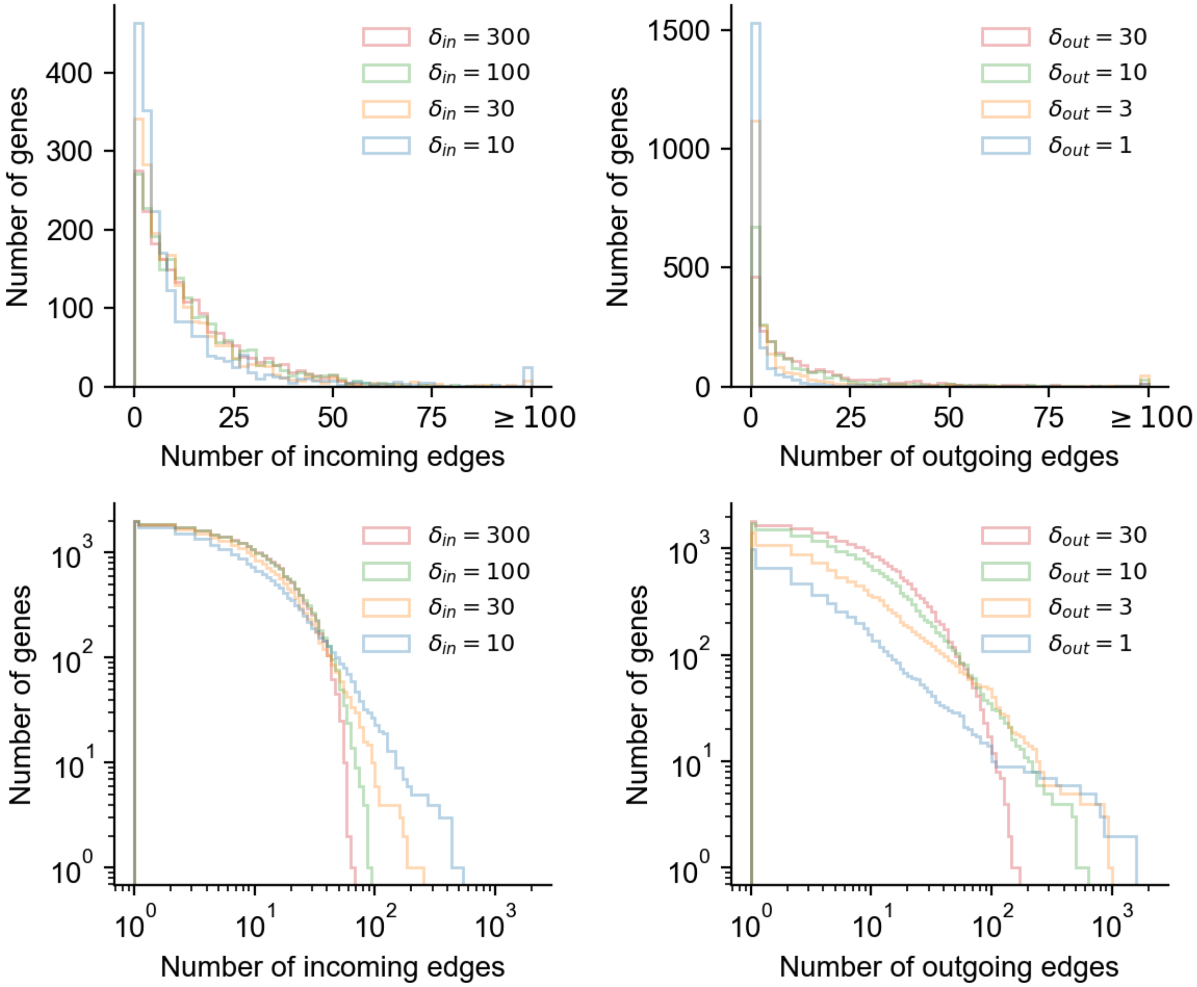

5. Fig. 3 analysis shows results for 50 GRNs. How were these 50 GRNs selected? Why not use all ~2000 or so?

This is a random downsample of GRNs in the study – the visualizations serve to give readers a sense of the orders of magnitude of variance in the study, across networks (3C), as a function of distance within networks (3D), and due to modularity (3E). The interpretations of these figures do not change if the underlying networks are resampled. This downsampling serves to avoid overplotting Figs. 3C and 3E, and helps to alleviate a cumbersome data slicing operation required to produce Fig. 3D.

6. Fig. 6A/B violin plot blue is hard to distinguish from gray boxplot interquartile ranges.

We have decreased the opacity of the violin plot in the figure. The updated Figure 6 is below.

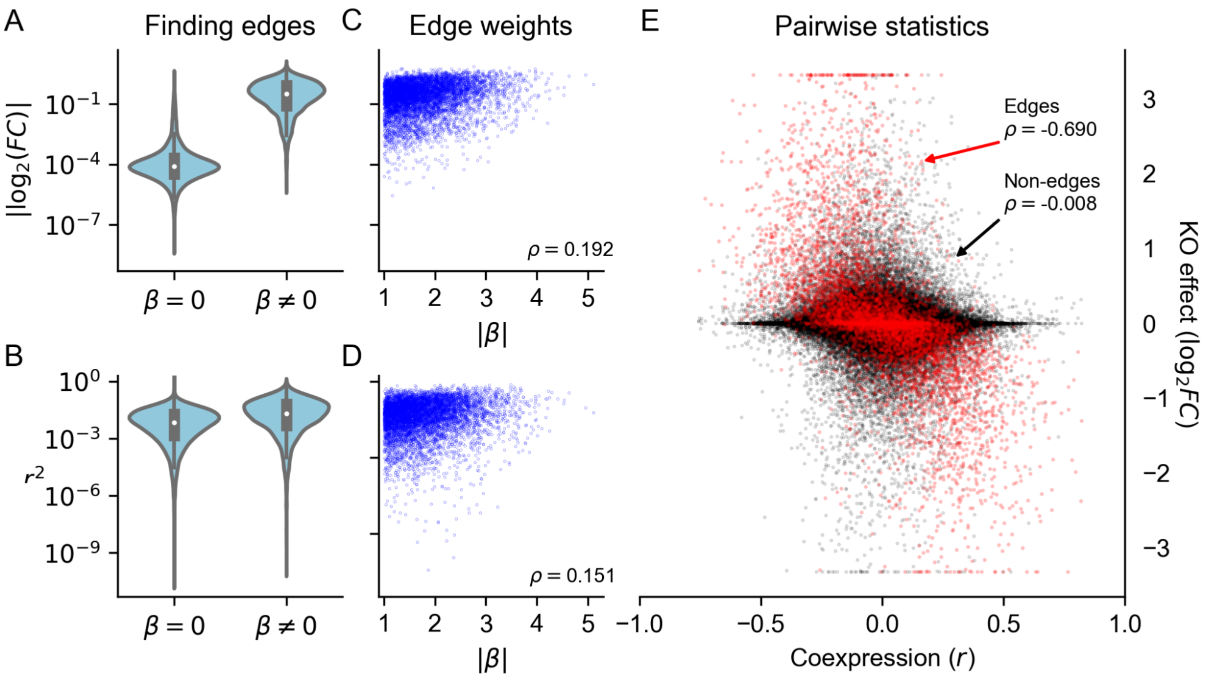

7. Fig. 7, add to legend how many genes per program are shown. In Methods, the K562 screen 100 genes per program are used, but Gene count is 200 in 7B, so not sure what number was used.

We thank the editor for noting this oversight and have added this quantification to the figure. The numbers in the figure were correct – we defined programs of 100 genes using the data from Replogle *et. al.*, but increased this to 200 for the synthetic network to match the size of its true groups. We have updated the analysis to keep the program size (200 genes) consistent between the real and simulated datasets, and have made corresponding edits to the Methods section. The updated results are identical in interpretation to the results of the previous version of the analysis. The below main and supplementary figures have been updated and are below (Figure S19, 7):

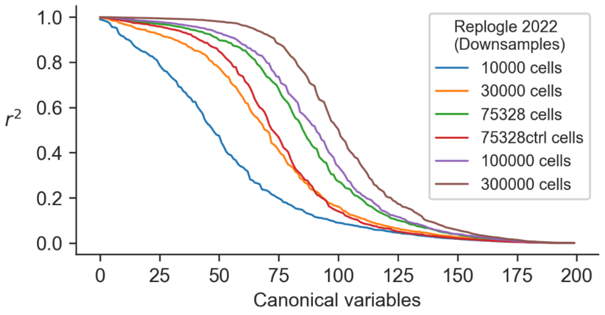

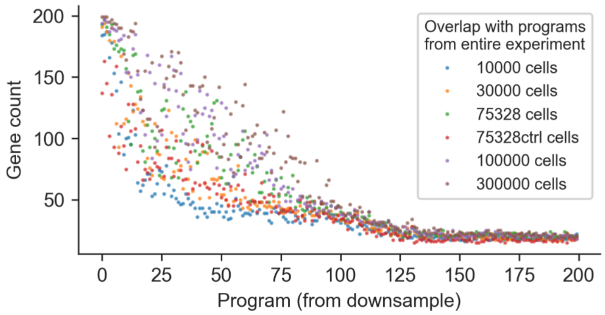

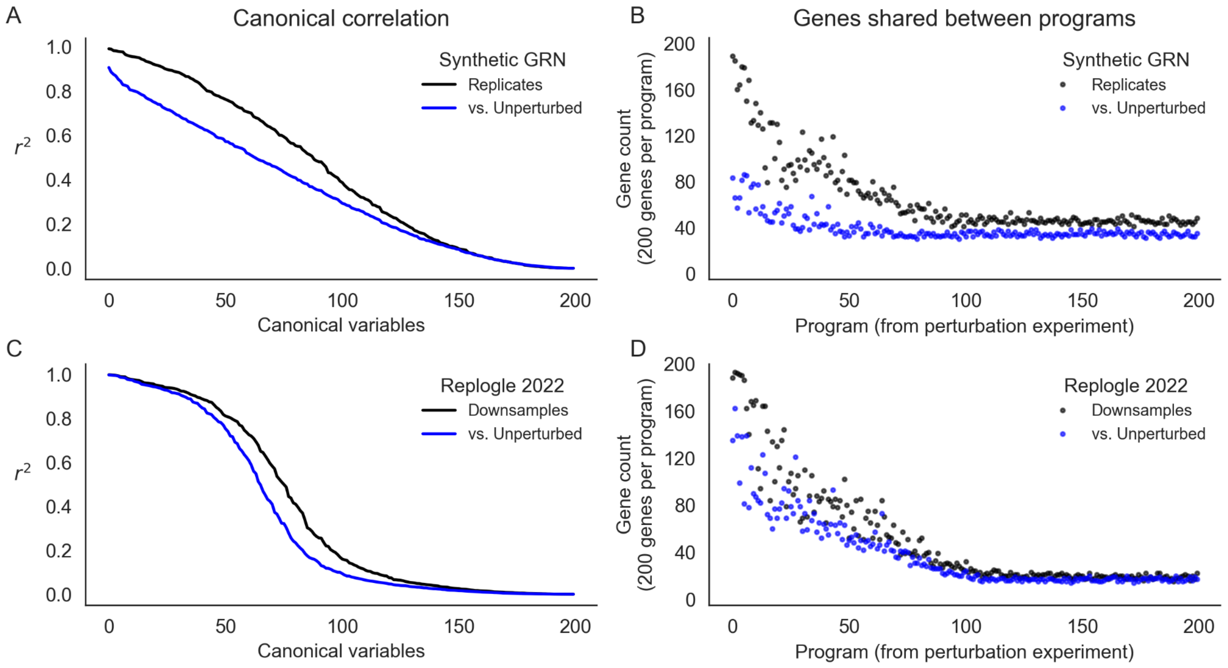

8. Fig. S1 would benefit from grid lines and a mark at log2(FC) = .1, the cutoff used to define hub KOs and their targets. How were 50 GRNs selected for inclusion in the figure? (Why not show all?)

We have added these visual elements to the Supplementary Figure. It is rather computationally intensive to plot the 7.7 billion gene pairs from all GRNs, so we used a downsample. For consistency, the included 50 GRNs are the same random sample shown in Fig. 3C. The updated figure (now S2) is below.

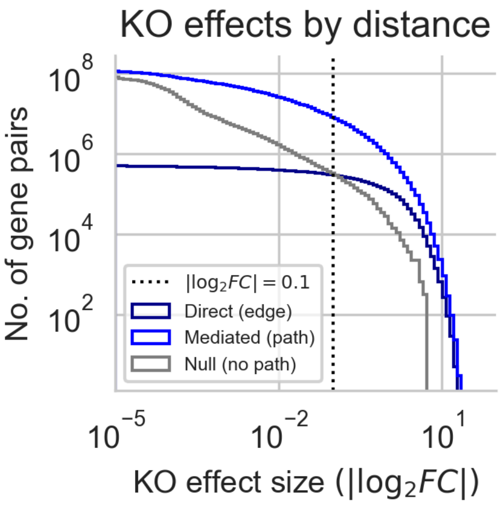

9. Fig. S3 and S4, please include full descriptions of the results shown rather than having reader refer back to Fig. 4 legend.

We have expanded the legends of these figures, and note that they are now Figs. S4 and S5 in the revised manuscript. We have similarly expanded the legends of Figs. S6 and S7. The legends are below:

Figure S4: Network generating parameters affect the number of KO effects. Counts of the number of perturbation effects per gene in the GRN with | log2 FC | ≥ 0.1 in each synthetic GRN, as a function of network generating parameters. Each panel shows all 1,920 GRNs as individual points, stratified by parameter values. Each distribution is annotated with its mean over GRNs (diamond points). We observe a similar direction of effect for each parameter as with the statistics presented in Figure 4.

Figure S5: Network generating parameters affect the stability of the fixed point. Counts of the number of genes which are hub targets in each synthetic GRN, as a function of network generating parameters, and stratifying by whether the expression equilibrium point of the synthetic GRN is stable (Methods). In all, 1,693 of the 1,920 GRNs (88.2%) reach an expression equilibrium through forward simulation of the SDE which is a stable fixed point of the corresponding ODE. These GRNs tend to be sparse (lower 1/p), modular (higher w), and have more hub regulators (lower δ_{out}), consistent with the direction of effect on the number of strong KOs and key target genes (as in Figure 4)

Reviewers' comments:

Reviewer's Responses to Questions

Comments to the Authors:

Reviewer #1:

I want to thank the authors for the detailed explanation for my concerns.

I still have some questions about how to connect synthetic network to real world data.

1. How you identify a synthetic GRN that is similiar to the pertub-seq data. In which criteria?

As stated in Methods (Section 4.3.2, “Perturb-seq data > Comparing with simulations”), we

> “compared the distributions of incoming and outgoing perturbation effects using the Kolmogorov-Smirnov test”

Moreover, these distributions of outgoing and incoming effects were computed at the gene level – for each gene, we counted how many genes it affects when perturbed (“outgoing effects”) and how many other gene perturbations affect its expression (“incoming effects”). Since the experimental data are subject to quality control and have a known but finite discovery rate, we do our best to match data from the simulated GRNs as if it were ascertained in a similar way. We do this by removing lowly expressed genes from the simulated data and by matching the genome-wide discovery rate to the experimental data by setting at a critical value of log fold-change. Additional details on this procedure are present in the same section of Methods (4.3.2), which we now reference in the corresponding section of the main text (Section 2.6, “Comparing with experimental data”).

2. How you pick the subset of the perturbations? What are those genes?

In the simulated GRNs, we perturb all genes and pick a subset of genes to analyze as above. In the genome-wide perturbation study from *Replogle et. al.*, we state in Section 2.1 (“Modeling approach”) that

> “we subset these data to 5,247 perturbations that target genes whose expression is also measured in the data”

This set of 5,247 genes corresponds to all genes which were both perturbed and measured by *Replogle et. al.* – roughly, these are all genes that are actively expressed in the K562 cell line. The data processing procedure is more fully described in Section 4.3 (“Perturb-seq data”).

3. Which kind of inference the method proposed can do? Can they infer TF-gene regulation?

We thank the reviewer for this forward-thinking question. We would like to clarify that our work describes a model to simulate gene expression under a variety of network structures (i.e., causal graphs) and conditions (i.e., perturbed and unperturbed states). In this paper, we characterize the parameters of the model and show how to use it to understand properties of real gene regulatory networks (namely sparsity, modularity, and properties of the degree distribution).

At the same time, we anticipate that our model can be applied towards various inferential tasks. For example, we imagine that synthetic data from our model (paired with ground truth observations) can be used to more rigorously benchmark existing inference algorithms; these synthetic data could also be

useful to augment the development and training of such algorithms. An example, following the type of analysis we performed in Figure 5, could be to estimate a prior distribution on network parameters (e.g., the number of regulators per gene) that could be used to narrow the search space of a network reconstruction algorithm. We briefly mention the potential uses of our approach in the Discussion section:

> "While the approach outlined in this work is not optimized for inference, the algorithms we describe are generative, which means that they could be used directly in applications for simulation-based inference"

We have added an example of how to use our results to modify approaches to network inference in Figure S18 (copied below). We have updated the Methods section with details on the implementation of this procedure, and have noted this analysis at the end of Section 2.7.1 of the Results ("Discovering pairwise relationships"),

> "As a simple example, we show that biasing the rank ordering of perturbation effect p-values from the Replogle et. al. dataset (to encourage edges to be drawn out from putative master regulators) improves correspondence with pairwise gene links from external sources on protein-protein and ChIP-seq interactions."

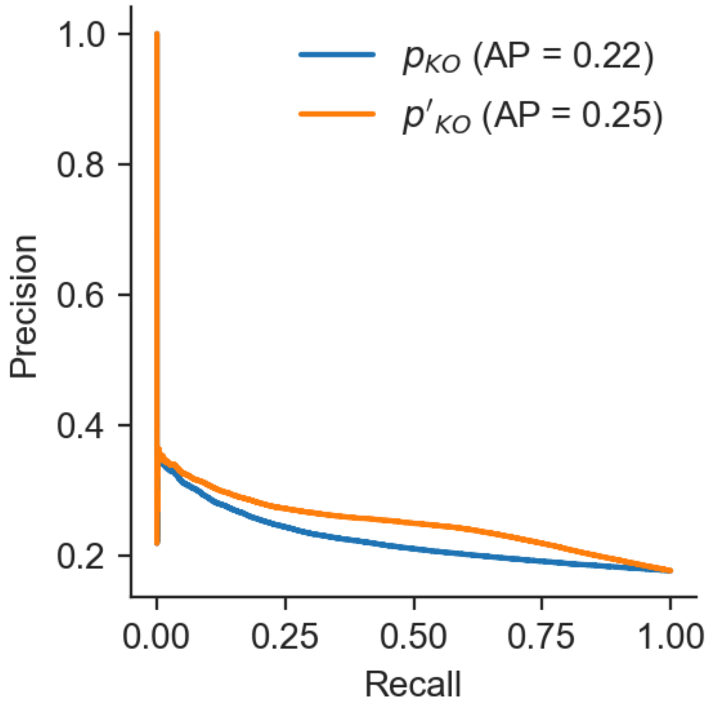

Finally, we have also made our analysis code available as a resource to the scientific community to work on applications of our methods. It can be found on github (https://github.com/maguirre1/grn-paper/).

Reviewer #2: The authors have adequately addressed my comments.

Reviewer #3: recommend to accept for publication.

Data Availability

Have the authors made all data and (if applicable) computational code underlying the findings in their manuscript fully available?

The PLOS Data policy requires authors to make all data and code underlying the findings described in their manuscript fully available without restriction, with rare exception (please refer to the Data Availability Statement in the manuscript PDF file). The data and code should be provided as part of the manuscript or its supporting information, or deposited to a public repository. For example, in addition to summary statistics, the data points behind means, medians and variance measures should be available. If there are restrictions on publicly sharing data or code —e.g. participant privacy or use of data from a third party—those must be specified.

Reviewer #1: None

Reviewer #2: Yes

Reviewer #3: Yes

Reviewer Identity

PLOS authors have the option to publish the peer review history of their article (what does this mean?). If published, this will include your full peer review and any attached files.

If you choose “no”, your identity will remain anonymous but your review may still be made public.

Do you want your identity to be public for this peer review? For information about this choice, including consent withdrawal, please see our Privacy Policy.

Reviewer #1: No

Reviewer #2: No

Reviewer #3: Yes: Zhana Duren

[NOTE: If reviewer comments were submitted as an attachment file, they will be attached to this email and accessible via the submission site. Please log into your account, locate the manuscript record, and check for the action link "View Attachments". If this link does not appear, there are no attachment files.]

Figure resubmission:

While revising your submission, please upload your figure files to the Preflight Analysis and Conversion Engine (PACE) digital diagnostic tool, https://pacev2.apexcovantage.com/. PACE helps ensure that figures meet PLOS requirements. To use PACE, you must first register as a user. Registration is free. Then, login and navigate to the UPLOAD tab, where you will find detailed instructions on how to use the tool. If you encounter any issues or have any questions when using PACE, please email PLOS at figures@plos.org. Please note that Supporting Information files do not need this step. If there are other versions of figure files still present in your submission file inventory at resubmission, please replace them with the PACE-processed versions.

Reproducibility:

To enhance the reproducibility of your results, we recommend that authors of applicable studies deposit laboratory protocols in protocols.io, where a protocol can be assigned its own identifier (DOI) such that it can be cited independently in the future. Additionally, PLOS ONE offers an option to publish peer-reviewed clinical study protocols. Read more information on sharing protocols at https://plos.org/protocols?utm_medium=editorial-email&utm_source=authorletters&utm_campaign=protocols

---

In compliance with data protection regulations, you may request that we remove your personal registration details at any time. (Remove my information/details). Please contact the publication office if you have any questions.
